# Synergistic Design of Flexible Nanopapers for High-Performance Proton Pseudocapacitors

**DOI:** 10.1007/s40820-025-01989-6

**Published:** 2026-01-05

**Authors:** Jiayue Dong, Zhaoqing Lu, Li Hua, Zizhan Guo, Xiaoxu Xu, Jinlong Wu, Fengfeng Jia, Yuanming Wang

**Affiliations:** 1 College of Bioresources Chemical and Materials Engineering, Shaanxi Provincial Key Laboratory of Papermaking Technology and Specialty Paper Development, National Demonstration Center for Experimental Light Chemistry Engineering Education, Shaanxi University of Science and Technology, Xi’an, 710021 People’s Republic of China; 2 Shaanxi ParaDe Advanced Material Technology Co., LTD, Building 5, Western Life Science Park, Fengdong New District, Xixian New Area, Xianyang, 712000 People’s Republic of China

**Keywords:** MXene, Graphene, Spatial architecture, Surface chemistry, Proton-type pseudocapacitor

## Abstract

Two-dimensional materials for flexible energy storage commonly face huge challenges in limited active surface and hindered charge transport. Herein, we report an innovative asymmetric pseudocapacitor based on synergistic design of modified MXene and graphene, integrating gas-induced rapid expansion technology and precise surface chemical regulation methods. For graphene modification, rapid vaporization induces exfoliation and expansion of graphene oxide layers. Subsequently, pseudocapacitive oxygen-containing groups were selectively introduced through acid oxidation, yielding expanded-and-oxidized graphene (OEG) for positive porous-nanopaper electrode. For MXene modification, alkali-treated MXene underwent hydrazine assistance to facilitate gas expansion and –NH_2_ grafting, producing MXene-NH_2_ (NOM) for negative porous-nanopaper electrode. Density functional theory calculations show that –COOH more effectively modulate graphene’s electronic structure by inducing charge redistribution and creating active sites, thereby enhancing H^+^ adsorption and ion interactions compared to –OH. Meanwhile, –NH_2_ on MXene enable electron delocalization and dynamic Ti–N–H^+^ interactions, speeding up proton adsorption/desorption and boosting both pseudocapacitance and conductivity. Through collaborative optimized spatial architecture and surface properties, flexible OEGB and NOMB exhibited of 333.6 and 500.5 F g^−1^ at high mass loading, respectively. The assembled proton pseudocapacitor readily achieved energy and power densities of 58.9 Wh kg^−1^ and 3802 W kg^−1^, respectively, with excellent stability for potential applications.

**Supplementary Information:**

The online version contains supplementary material available at 10.1007/s40820-025-01989-6.

## Highlights

- By utilizing water vaporization to increase the surface area of graphene and precisely controlling the ratio of oxygen-containing functional groups, the optimal –COOH:–OH ratio of 1:1 was successfully achieved, resulting in a maximum pseudocapacitance of 430.5 F g^−1^.
- Through hydrazine-assisted hydrothermal reaction, –F groups on the MXene surface were substituted with –NH_2_, while gas generation facilitated the creation of a porous structure, boosting the capacitance to 500.5 F g^−1^ under high mass loading conditions.

**Supplementary Information:**

The online version contains supplementary material available at 10.1007/s40820-025-01989-6.

## Graphical abstract

**Figure:**
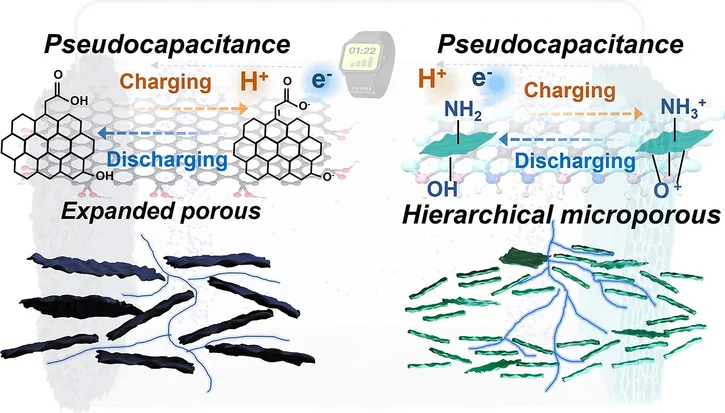

## Introduction

Flexible supercapacitors have garnered significant attention due to their broad applications in wearable and portable electronic devices [1–3]. Among various candidate materials, two-dimensional (2D) materials—such as graphene, MXenes, black phosphorus, and covalent organic frameworks (COFs)—stand out owing to their exceptional flexibility, high packing density, and strong interlayer interactions, demonstrating huge potential [4–8]. As an emerging 2D material, MXene has the booming development for high-performance flexible supercapacitors, particularly in proton-type supercapacitors [9]. Proton-type supercapacitors leverage smallest ionic radius and fast diffusion kinetics, enabling outstanding energy storage performance in solid-state systems, and represent a critical pathway toward achieving high-rate capability and high-energy density [10, 11]. Notably, MXene exhibits excellent specific capacitance and stability in acidic environments, effectively highlighting its tremendous potential as a proton-type negative electrode material [12]. However, 2D materials commonly suffer from severe interlayer restacking under high mass loading, resulting in insufficient exposure of active sites and increased ion diffusion path tortuosity [13]. Thus, materials optimization remains a critical issue that urgently needs to be addressed [14, 15]. Meanwhile, although MXene-based flexible supercapacitors have achieved relatively high energy density via asymmetric configurations, this approach is limited by the lack of efficient and performance-matched 2D positive electrode materials. To our knowledge, no one is more suitable as a positive electrode material than graphene to match with MXene, given that 2D carbon materials have better stability and voltage window at the same acid condition [16]. However, graphene’s energy storage is predominantly governed by electric double-layer capacitance, with limited pseudocapacitive contribution and relatively low capacitance, which hinders full utilization of asymmetric system potential [17–19]. Therefore, the development of a proton-type solid-state asymmetric supercapacitor employing a high-pseudocapacitance 2D MXene negative electrode and a well-matched pseudocapacitive 2D graphene positive electrode is considerably attractive and meaningful [20]. It is believed to achieve such flexible energy storage devices with high electrochemical performance and mechanical flexibility, thereby meeting the demands of next-generation portable and wearable energy systems.

To overcome critical issues in 2D materials such as less electrochemically active surface, restricted ion diffusion and severe capacitance degradation in high mass loading electrodes, researchers have proposed a variety of strategies [21–23]. Among them, spatial architecture design and regulation of electrochemically active surface functional groups are widely regarded as one of the two most effective modification approaches [24–26]. On one hand, constructing meso-/macrospatial architectures within 2D layered materials can significantly enhance electrolyte accessibility and alleviate ion transport limitations, especially in thick electrode systems [27–29]. For example, techniques such as freeze-drying and template etching have been extensively used to fabricate porous networks in graphene and MXene, thereby effectively boosting ion diffusion rates and interfacial reaction kinetics [30–32]. However, it should be noted that such spatial architectures in bulk or aerogel materials often limited the flexibility and volumetric energy density of the device. On the other hand, introducing redox-active surface functional groups can effectively improve material wettability and pseudocapacitive contributions [33]. However, introducing pseudocapacitive active groups on graphene remains significant challenging due to its inert carbon basal plane and limited surface chemistry activity. Functionalization often requires prolonged oxidation or harsh treatments that may damage the carbon framework, limiting graphene-based electrodes to specific capacitances below 300 F g^−1^ [34, 35]. In contrast, MXene naturally possesses surface groups like –O and –F, which can be chemically substituted with more electrochemically active groups such as –OH and –SO_3_H under suitable conditions, improving hydrophilicity, proton adsorption, and pseudocapacitive activity [36–38]. Despite these modifications, the electrochemical potential of MXene remains underutilized, indicating the necessity for continued exploration and innovative strategies to further enhance its performance. In this context, the synergistic combination of spatial structure design to accelerate ion transport and highly active surface functional groups to enhance pseudocapacitance is believed to be an effective strategy to overcome the performance limitations of 2D materials.

To address these critical limitations, we report an advanced asymmetric proton-type pseudocapacitor based on the rational integration of modified MXene and graphene electrodes, achieved through a dual strategy of gas-driven expansion and precise surface chemistry regulation. For the positive electrode, we employed a rapid water-evaporation-induced exfoliation and expansion strategy to achieve in situ delamination and structural expansion of graphene oxide (GO), significantly exposing more electrochemically active sites. Subsequently, a high-efficiency mixed acid oxidation was used to precisely introduce –OH and –COOH functional groups, enabling controlled distribution of the surface functionalities. Combined with experimental results and DFT calculations [39], we systematically revealed that –COOH groups in graphene more effectively modulate the electronic structure and enhance H^+^ adsorption, explaining why OEGB exhibits optimal performance when the –COOH:–OH ratio approaches 1:1. For the negative electrode, KOH pre-treatment was used to regulate the surface terminal groups of MXene and introduce –OH, followed by a hydrazine-assisted hydrothermal reaction to achieve efficient grafting of –NH_2_ functional groups, accompanied by gas-induced expansion to form a porous structure. The –NH_2_ groups in MXene promote electron delocalization and dynamic Ti–N–H^+^ interactions, accelerating proton adsorption/desorption and enhancing conductivity. We further revealed the stabilization mechanism of MXene via the –OH to μ_3_–O transformation during H^+^ adsorption, as well as the superior electronic properties of –NH_2_ compared to –OH, which are consistent with the electrochemical results. This design fully demonstrates the synergistic optimization from molecular-level functional groups to macroscopic porous structures, achieving high performance and structural stability in both positive and negative electrodes. The resulting OEG/BC (OEGB) and NOM/BC (NOMB) porous nanopaper achieves a specific capacitance of 333.6 and 500.5 F g^−1^ at high mass loading, respectively. Benefiting from the synergistic optimization of spatial structure and surface chemistry, as well as the high matching degree between the positive and negative electrodes, the fabricated electrode pair maintains excellent electrochemical performance even at a total mass loading of 10 mg cm^−2^. The assembled asymmetric supercapacitor delivers a high energy density of 58.9 Wh kg^−1^ and a power density of 3802 W kg^−1^, along with an outstanding capacitance retention of 89.7% after 30,000 cycles. These results clearly demonstrate the great potential of rationally designed 2D thick electrodes in flexible energy storage.

## Experimental Section

### Materials

The MXene precursor Ti₃C₂Al was provided by Shengxene Technology Co., Ltd. (China), and graphene oxide powder was purchased from Kery Nano Co., Ltd. (China). LiF was sourced from Shanghai Mclin Biochemical Technology Co., Ltd. (China). Bacterial cellulose was supplied by Hainan Yide Food Co., Ltd. (China). Other chemical reagents and materials, including concentrated sulfuric acid (H₂SO₄, ≥ 98 wt%) and hydrochloric acid (HCl, 35 wt%), were procured from Sinopharm Chemical Reagent Co., Ltd. (China).

### Anode OMB and NOMB Nanopaper

#### Preparation of OMB Nanopaper

MXene and KOH were blended at varying mass ratios (1:0, 1:1, 1:2) and magnetically stirred at room temperature for 8 h to obtain MXene-OH suspensions. Subsequently, the resulting suspensions were mixed with bacterial cellulose (BC) at a mass ratio of 3:2. The OMB composite nanopapers were fabricated via vacuum-assisted filtration through a 0.22-μm microporous film, followed by thermostatic vacuum drying at 105 °C. Based on the MXene/KOH ratios, the nanopapers were designated as OMB_0_, OMB_1_, and OMB_2_, respectively. The active material loading is 5 mg cm^−2^, while the BC loading is 3.3 mg cm^−2^.

#### Preparation of NOMB Nanopaper

The OMB nanopaper was lay on the surface of a first alumina ceramic substrate pre-treated with 300 μL of hydrazine hydrate solution. After complete wetting of the nanopaper by the solution, a second alumina ceramic substrate was overlaid to form a sandwich-structured intermediate. This assembly was transferred into a sealed culture dish and thermally cured in a vacuum oven at 90 °C for 5 h, yielding the NOMB nanopaper. Depending on the varied mass ratios of MXene to KOH, the resultant composite nanopapers were designated as NOMB_0_, NOMB_1_, and NOMB_2_, respectively. The active material loading is 5 mg cm^−2^, while the BC loading is 3.3 mg cm^−2^.

### Preparation of Cathode EGB and OEGB Nanopaper

#### Preparation of EGB Nanopaper

Mix deionized water with GO powder and continuously stir to promote the evaporation of deionized water, obtaining a GO dough. Place the GO dough between two polytetrafluoroethylene (PTFE) films and transfer it into a cold press machine for shaping to obtain a GO film. Place the GO film in a sealed aluminum container and heat it using an alcohol lamp. The rapid high-temperature vaporization of water molecules induces expansion and reduction reactions, yielding expanded graphene (EG) powder. Finally, the EG powder was mixed with BC at a mass ratio of 4:1 and processed through vacuum-assisted filtration followed by freeze-drying to obtain the EGB nanopaper. The active material loading is 3 mg cm^−2^, while the BC loading is 0.75 mg cm^−2^.

#### Preparation of OEGB Nanopaper

The sulfuric acid and nitric acid were mixed at a volume ratio of 3:1 to obtain a mixed acid solution. EG powder was then added to the mixed acid solution at a concentration of 3 mg mL^−1^. After stirring at 0 °C to obtain OEG powder, the product was mixed with BC at a mass ratio of 4:1 and subjected to vacuum filtration to form the OEGB nanopaper. The resultant composite nanopapers were designated as OEGB-4 h, OEGB-6 h, and OEGB-8 h based on their respective reaction durations (4, 6, and 8 h) under continuous agitation. The active material loading is 3 mg cm^−2^, while the BC loading is 0.75 mg cm^−2^.

### Preparation of PAM/H_2_SO_4_ Gel Electrolytes

PAM/H_2_SO_4_ gel electrolyte was prepared by dissolving 2 g of acrylamide in 10 mL of 1 M H_2_SO_4_ solution. Then, 4 mg of N, N′-methylenebisacrylamide (MBAA) and 5 mg of ammonium persulfate (APS) were added sequentially under continuous stirring until fully dissolved. The mixture was poured into a mold and cured in an oven at 60 °C for 1 h to obtain the PAM/H_2_SO_4_ gel electrolyte.

### Fabrication of the Solid-State NOMB//OEGB Asymmetric Device

A solid-state asymmetric supercapacitor was assembled using NOMB as the negative electrode and OEGB as the positive electrode, with a PAM/ H_2_SO_4_ gel directly serving as both the separator and electrolyte between the two electrodes. A 0.5 cm length at the end of each electrode was left uncoated with the gel electrolyte to ensure effective electrical contact, which was established using highly conductive graphene paper. To eliminate charge imbalance, the active material loadings and electrode areas of the positive and negative electrodes were carefully adjusted. For NOMB, the loading was 5 mg cm^−2^ with an electrode area of 0.8 cm^2^, while for OEGB, the loading was 5 mg cm^−2^ with an electrode area of 1.2 cm^2^.

## Results and Discussion

**Fig. 1:**
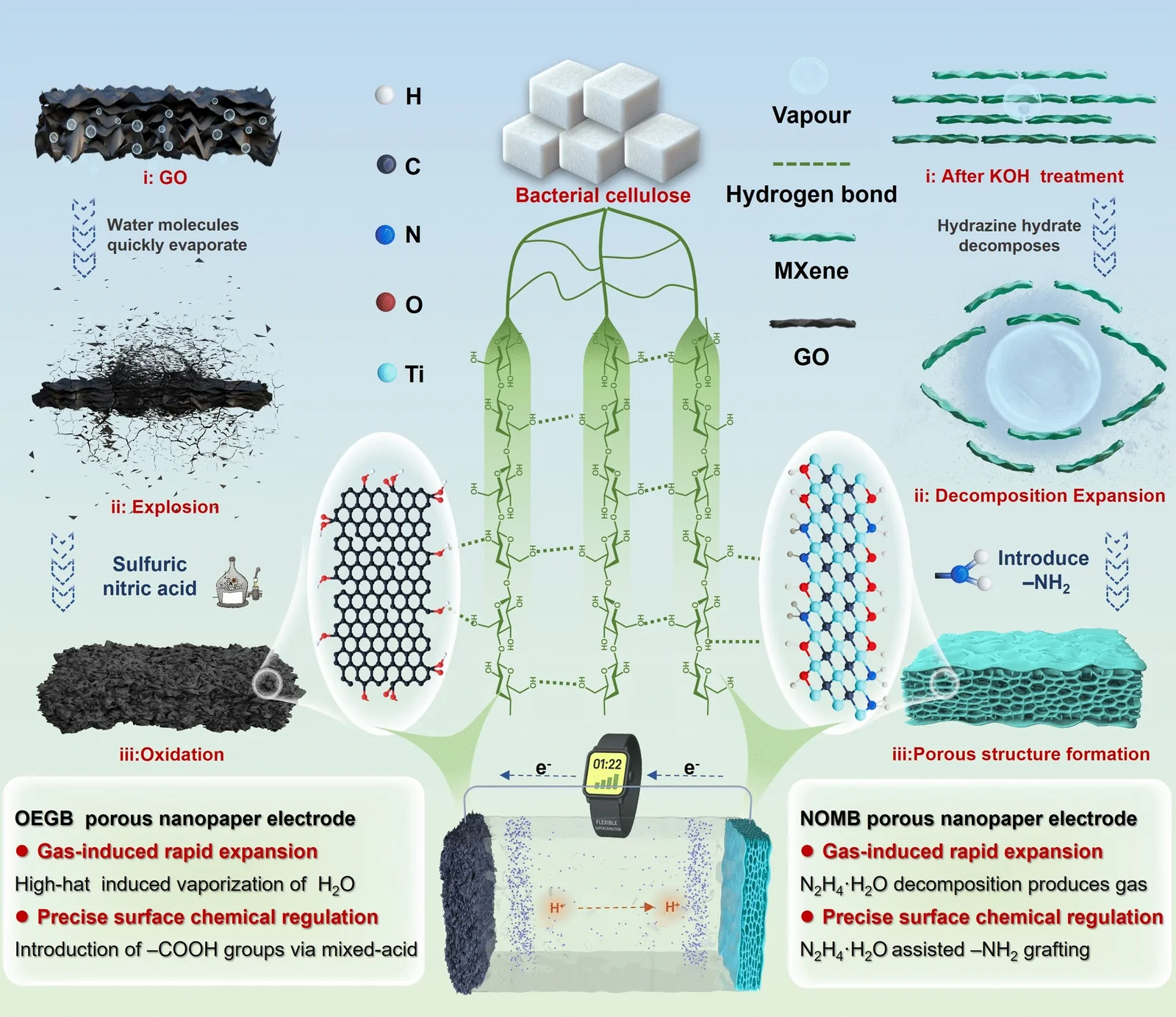
Preparation principle of OEGB and NOMB nanopaper electrodes

As illustrated in Fig. 1, we constructed a novel asymmetric pseudocapacitor based on synergistic design of MXene and graphene, integrating gas-induced rapid expansion technology and precise surface chemical regulation methods. For the positive electrode, GO was dispersed in deionized water and processed via a kneading-and-laminating method, inspired by the traditional Chinese noodle-making technique. Explosive vaporization of water molecules upon rapid heating effectively disrupted the π–π stacking interactions among GO layers, resulting in internal rupture and the formation of expanded graphene (EG). EG was further oxidized using a mixed acid treatment to selectively modify the surface chemistry, yielding expanded-and-oxidized graphene (OEG). For the negative electrode, the precusor was based on our previous work on KOH-treated MXene with optimized surface terminals and chemical environment [40]. Subsequently, during the hydrazine hydrothermal reaction, hydrazine decomposed and generated gas, which expanded the interlayer spacing and formed a hierarchical microporous structure. Concurrently, alkaline conditions facilitated the substitution of inert MXene surface groups by –NH_2_, resulting in chemical functionalization. In addition, we introduce natural bacterial cellulose (BC) into the positive and negative electrode to enhance the hydrophilicity of the electrode materials and improve the compatibility between the electrode and the electrolyte [41, 42]. This not only effectively prevents the active material from detaching during charge and discharge cycles but also strengthens the structure by forming hydrogen bonds and other interactions with the active material, thereby improving its stability and strength.

### Positive Electrode

#### Materials Characterization

**Fig. 2:**
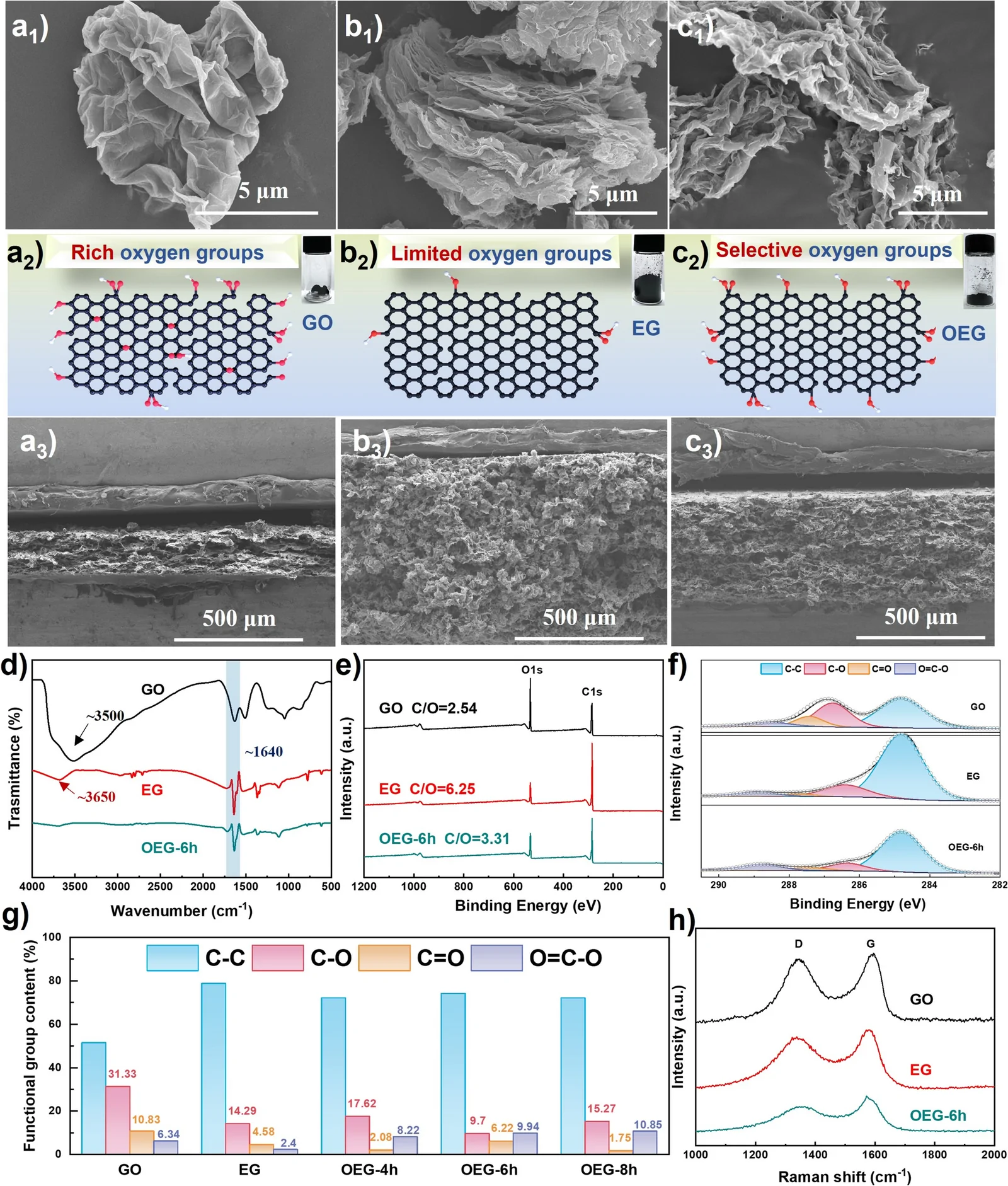
Morphology and chemical characterization of GO, EG, and OEG powders, and their composite nanopapers with BC. SEM image of **a**_**1**_ GO powder; **b**_**1**_ EG powder; **c**_**1**_ OEG-6 h powder; schematic illustration of the chemical structure: **a**_**2**_ GO powder; **b**_**2**_ EG powder; **c**_**2**_ OEG-6 h powder (top right of photograph of 100 mg powder samples for visual comparison); Cross-sectional SEM images of BC composite nanopapers: **a**_**3**_ GOB nanopaper; **b**_**3**_ EGB nanopaper; **c**_**3**_ OEGB-6 h nanopaper; Chemical characterization of GO, EG, and OEG powders at different oxidation durations: **d** FTIR spectra; **e** XPS survey spectra;**f** high-resolution XPS C 1*s* spectra; **g** relative content of carbon bonds derived from C 1*s* spectra; **h** Raman spectra

For the positive electrode, Fig. 2a1–c1 displays the scanning electron microscopy (SEM) images of GO, expanded graphene (EG), and expanded-and-oxidized graphene (OEG) powders, respectively. The GO powder exhibits pronounced aggregation. In contrast, the EG powder, prepared via rapid high-temperature water vapor-induced expansion, displays an accordion-like layered morphology reminiscent of MXene. After further oxidation, the OEG powder shows distinct wrinkling of the sheets. The optical images in the upper-right corners of Fig. 2a2–c2 clearly show that, at the same mass, the powder volumes decrease in the order EG > OEG > GO. This trend can be mainly attributed to the morphological transformations induced by variations in surface functional groups, indicating that rapid water vapor expansion not only tailors the microstructure of graphene powders but also markedly enhances their specific surface area. Specifically, the BET results (Fig. S1a–d) show that GO exhibits a surface area of only 47.95 m^2^ g^−1^, while EG dramatically increases to 349.26 m^2^ g^−1^ after rapid expansion. Upon further regulation of surface functional groups, OEG displays a surface area of 123.24 m^2^ g^−1^. These results collectively verify the effectiveness of rapid water vapor expansion in optimizing the pore structure and enlarging the specific surface area of graphene powders. Following vacuum filtration and freeze-drying, GO, EGB, and OEGB nanopaper electrodes were fabricated from the respective powders, and their microstructural features were largely preserved. Cross-sectional SEM images of the GOB, EGB, and OEGB nanopapers are shown in Fig. 2a3–c3. A variation in thickness is observed, with the EGB nanopaper exhibiting the greatest thickness due to fragmented structures. Additionally, cross-sectional analysis of nanopapers with different oxidation times (Fig. S1e–h) reveals that the nanopaper thickness decreases with prolonged oxidation. This is because the acid solvent used during the oxidation process may affect the interlayer structure of EG, disrupting its original loose structure, leading to the removal of interlayer moisture or structural changes, thereby reducing the thickness.

Figures 2d and S1i show the Fourier transform infrared (FTIR) spectra of GO, EG, and OEG powders with different oxidation durations. A broad O–H stretching vibration peak is clearly observed around 3500 cm^−1^ in the GO powder. After high-temperature expansion, the intensity of this peak significantly decreases, indicating the thermal decomposition of some –OH groups. With increasing oxidation time, the intensity of the O–H peak gradually increases, indicating the reintroduction or regeneration of oxygen-containing functional groups. X-ray photoelectron spectroscopy (XPS) analyses (Figs. 2e–g and S1j–l) provided insights into surface chemistry changes. As shown in Figs. 2e and S1f, GO exhibits the lowest C/O atomic ratio (2.54) and the highest oxygen content, with C–O (~ 286.4 eV) and C=O (~ 287.9 eV) being dominant, while O–C=O (~ 288.9 eV) is relatively minor, indicating a highly oxidized state with a comparatively limited proportion of carboxyl groups [43]. After thermal expansion, the C/O ratio of EG rises to 6.25, suggesting decomposition of surface groups, carbon framework reconstruction and partial defect repair, leading to a more stable graphene structure. This is further confirmed by the significant increase in the C–C peak intensity of EG in the high-resolution XPS C 1*s* spectra (Fig. 2f). Upon subsequent oxidation, the C/O ratio of OEG gradually decreases from 3.42 to 3.25, indicating the progressive reintroduction of oxygen functionalities, which helps enhance electrochemical activity. This is further confirmed by high-resolution XPS C 1*s* spectra (Fig. 2g). Additionally, the oxidation time serves as a key parameter for tuning the type and distribution of oxygen-containing functional groups. Based on XPS analysis, the following conclusions can be drawn: at 4 h (OEG–4 h), and the C–O content was 11.6% and O–C=O accounted for 8.2%, corresponding to an O–C=O/C–O ratio of 0.71, indicating that –OH groups still dominated, with only partial conversion to –COOH. After extending to 6 h (OEG–6 h), C–O decreased to 9.7%, while O–C=O increased to 9.9%, bringing the O–C=O/C–O ratio close to 1.0, clearly demonstrating that some –OH had been converted to –COOH, at which point the carboxyl content reached its peak. Further oxidation to 8 h (OEG–8 h) led to a further increase of O–C=O to 10.9%, while C–O rebounded to 15.3%, indicating regeneration of –OH groups. At this stage, the O–C=O/C–C ratio reached its highest value (0.15), reflecting excessive oxidation and over-functionalization on the surface, accompanied by graphene sheet fragmentation and structural deterioration. Raman spectra (Figs. 2h and S2) provide additional evidence for structural changes during oxidation. The intensity ratio of the D to G bands (I_D_/I_G_) increases with oxidation time (Table S1), confirming the rise in defect density and the transition toward an amorphous carbon structure under prolonged treatment. Therefore, tuning the oxidation time offers an effective strategy to control the surface functional group density on graphene, allowing for the selective introduction of oxygen functionalities that are favorable for pseudocapacitive behavior.

#### Electrochemical Performance

**Fig. 3:**
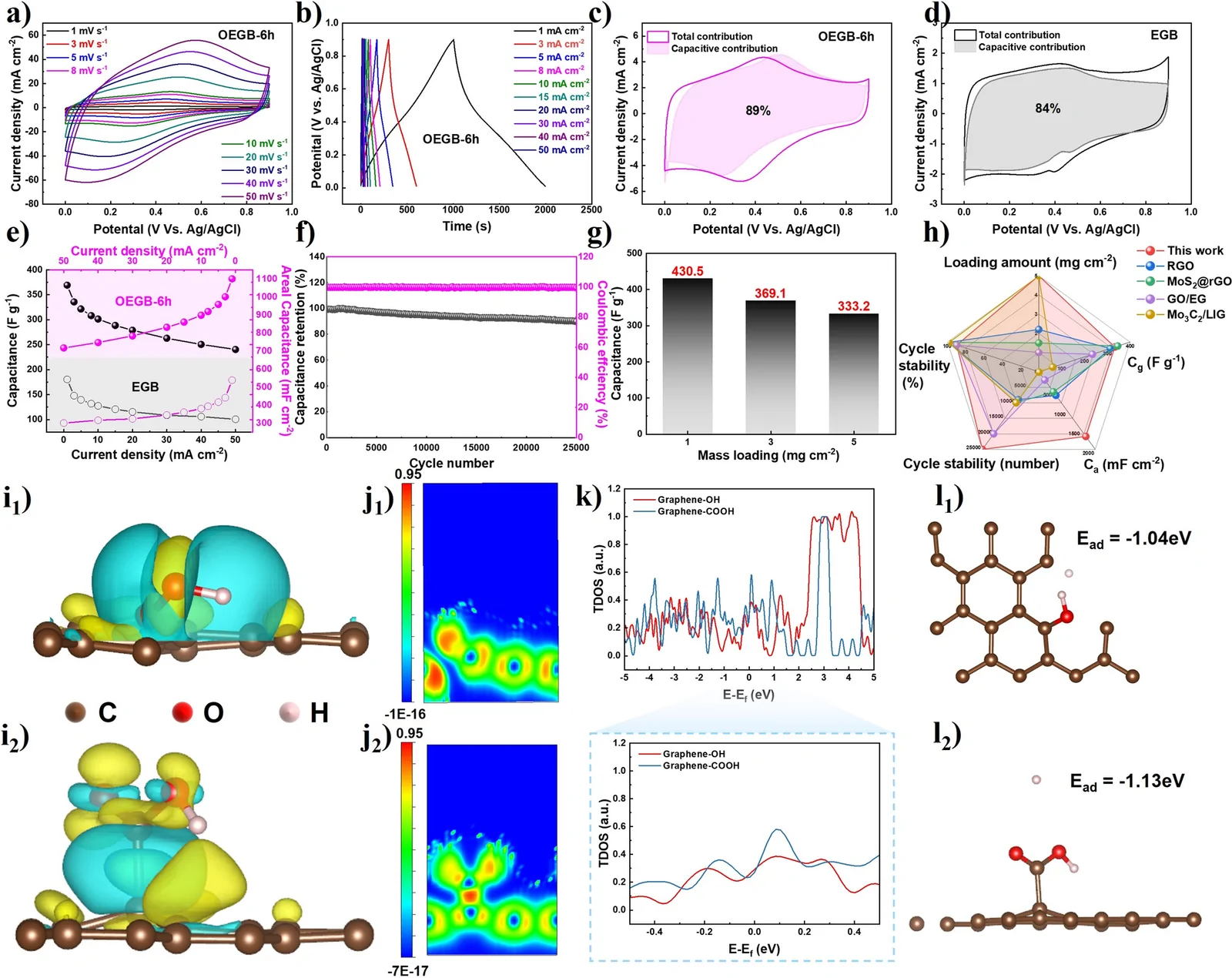
Electrochemical performance of EGB and OEGB–6 h composite nanopapers. **a** CV curves of the OEGB–6h nanopaper at different scan rates; **b** GCD curves of the OEGB–6h nanopaper at various current densities; **c** CV curve of the OEGB–6h nanopaper at 3 mV s^−1^, with the shaded area indicating the surface capacitive contribution; **d** CV curve of the EGB nanopaper at 3 mV s^−1^, with the shaded area indicating the surface capacitive contribution; **e** comparison of specific capacitance between EGB and OEGB–6h nanopapers; **f** cycling stability and corresponding coulombic efficiency of the OEGB–6h nanopaper at 20 mA cm^−2^; **g** specific capacitance of OEGB–6 h nanopaper under different mass loading was compared; **h** radar charts show the excellence of OEGB–6h nanopaper in all aspects of performance; Side views of differential charge density: **i**_**1**_ G–OH, **i**_**2**_ G–COOH; electron localization function (ELF) plots: **j**_**1**_ G–OH, **j**_**2**_ G–COOH; **k** comparison of total density of states (DOS) at the Fermi level between G–OH and G–COOH; optimized structures of H^+^ adsorption on G–OH (**l**_**1**_) and G–COOH (**l**_**2**_), along with their corresponding adsorption energies

To verify the feasibility of the proposed strategy, electrochemical performance tests were carried out. Figures 3a, b and S3a, b present the cyclic voltammetry (CV) curves and galvanostatic charge–discharge (GCD) curves of the EGB and OEGB–6 h nanopapers, respectively. It is clearly observed that the OEGB–6 h sample exhibits more pronounced redox peaks, consistent with the previous XPS analysis, indicating that the reintroduced oxygen-containing functional groups through secondary oxidation significantly enhance the pseudocapacitive contribution. The capacitive contribution analysis and fitting results are shown in Fig. 3c, d, confirming that the OEGB–6 h nanopaper indeed exhibits a higher pseudocapacitive contribution compared to the EGB nanopaper at the same current density. Figure 3e shows that, at a current density of 1 mA cm^−2^, the specific capacitance of the OEGB–6 h nanopaper reaches 369 F g^−1^ (1100 mF cm^−2^), approximately 2.04 times that of the EGB nanopaper (180 F g^−1^, 538 mF cm^−2^). The observed performance enhancement is primarily attributed to the synergistic effect between structural regulation and the selective introduction of surface functional groups. The expansion–oxidation process constructs a spatial architecture and introduces abundant pseudocapacitive active sites, which significantly shorten ion diffusion pathways and thereby result in superior pseudocapacitive behavior and overall electrochemical performance. Furthermore, electrochemical impedance spectroscopy (EIS) was employed to investigate the electrode/electrolyte interface behavior. Figure S3c, d shows the Nyquist plots and the fitting of Z' versus ω^−1/2^ in the low-frequency region. As seen in the high-frequency enlarged view, the EGB nanopaper exhibits a lower charge transfer resistance (R_ct_). After oxidation, the OEGB–6 h nanopaper shows a slight increase in Rct due to the incorporation of additional oxygen-containing groups, although the conductivity loss remains within an acceptable range (Fig. S3e). Notably, in the Warburg region of the Nyquist plot, the slope of the OEGB–6 h nanopaper is lower than that of the EGB nanopaper, suggesting a higher surface ion transport rate. Figure S3f, g presents the Bode plots and normalized capacitance spectra. The characteristic time constants corresponding to the phase angle peaks are approximately 21.9 ms for the EGB nanopaper and 21.7 ms for the OEGB–6 h nanopaper. The phase angle peak of the EGB nanopaper is closer to − 90°, indicating a behavior closer to that of an ideal capacitor. In contrast, the OEGB–6 h nanopaper exhibits a slower time constant in the low-frequency region of the normalized capacitance spectrum (τ₀ ≈ 739 ms), further reflecting its distinctive pseudocapacitive characteristics. Moreover, the OEGB–6 h nanopaper demonstrates excellent cycling stability, maintaining 89% of its initial capacitance after 25,000 charge–discharge cycles (Fig. 3f). In comparison, EGB retains 86.8% of its capacitance after 15,000 cycles (Fig. S3h).

Although GO contains abundant oxygen-containing functional groups, its electrochemical performance tends to degrade. This is primarily due to differences in oxidation time and the distribution of oxygen-containing groups on the surface. To control the content and distribution of these groups, we performed electrochemical analysis on samples treated with different oxidation times. Figure S4a–d shows the CV and GCD curves of samples oxidized for 4 h and 8 h under different scan rates and current densities. Figure S4e, f shows the CV and GCD curves at the same scan rate and current density for samples treated with different oxidation times. From these curves, it is evident that as the oxidation time increases (after 6 h), both the CV curve area and the GCD time decrease significantly. To further elucidate the influence of surface functional groups on the electrochemical performance of graphene, XPS analysis (Fig. 2g) was conducted to assess the chemical composition of samples subjected to different oxidation durations. The results reveal that the OEGB–6 h sample exhibits an approximately 1:1 ratio of –COOH to –OH functional groups, with the highest –COOH content and the lowest –OH content among all samples. In contrast, the OEGB–4 h sample primarily contains a small amount of –OH with limited –COOH content, while the OEGB–8 h sample suffers from structural damage and excessive –OH generation due to over–oxidation. These observations, when correlated with electrochemical measurements, indicate that OEGB–6 h delivers superior pseudocapacitive performance. This suggests that –COOH plays a more dominant role than –OH in enhancing electrochemical activity. Therefore, a moderately oxidized surface with a near 1:1 –COOH/–OH ratio, in which –COOH is more prevalent, is considered optimal for achieving a favorable balance between structural integrity and pseudocapacitive contribution. Figure S4g, h shows the mass-specific capacitance and area-specific capacitance of different nanopapers. At a current density of 1 mA cm^−2^, the OEGB nanopaper exhibited a mass-specific capacitance of 180 F g^−1^ (538 mF cm^−2^), the OEGB–4 h nanopaper 329 F g^−1^ (981 mF cm^−2^), the OEGB–6 h nanopaper 369 F g^−1^ (1100 mF cm^−2^), and the OEGB–8 h nanopaper 320 F g^−1^ (954 mF cm^−2^). To further evaluate the electrochemical performance of the OEGB electrode under practical conditions, systematic tests were conducted on samples with different mass loadings, as shown in Figs. 3g and S5. At a low mass loading of 1 mg cm^−2^, the OEGB–6 h electrode delivers a high specific capacitance of 430.5 F g^−1^ (430 mF cm^−2^), indicating excellent charge storage capability. Even at 5 mg cm^−2^ mass loading, the electrode retains typical pseudocapacitive behavior, with a specific capacitance of 333.6 F g^−1^ (1668 mF cm^−2^). These results demonstrate that the OEGB–6 h electrode exhibits outstanding electrochemical performance and stability across various loading conditions, indicating strong potential for practical applications (Fig. 3h).

#### Theoretical Calculation

The enhancement of supercapacitor performance relies on a profound understanding of electrode materials at the atomic scale. To this end, density functional theory (DFT), as an indispensable quantum mechanical tool, is widely employed to reveal the electronic structures and interaction mechanisms of materials. XPS results indicate that the OEGB–6 h nanofiber paper with the best electrochemical performance shows the largest difference in –OH and –COOH functional group contents compared to others. Therefore, we constructed graphene models functionalized with –OH and –COOH groups, respectively. We deliberately constructed graphene models functionalized solely with –COOH or –OH groups to distinguish their intrinsic contributions to electrochemical performance. This design allows us to clearly reveal the individual effects of each functional group and to confirm the advantage of –COOH in enhancing electrochemical performance. First, their differential charge density distributions were comparatively analyzed (Fig. 3i), where cyan regions represent electron depletion, and yellow regions indicate electron accumulation. The –OH group induces only localized and limited charge perturbations, exerting a relatively weaker influence on the overall electronic structure (Fig. 3i1). In the contrary, the introduction of –COOH groups induces a more pronounced electron redistribution, characterized by distinct regions of charge accumulation and depletion on the surface. This indicates a net electron transfer from the graphene substrate to the –COOH region, leading to an overall charge accumulation within the functional group. Although the two oxygen atoms in the –COOH group are highly electronegative, they do not explicitly appear to “gain” electrons in Fig. 3i2, as the differential charge density map reflects spatial charge redistribution rather than the net charge on individual atoms. However, Bader charge analysis confirms that each oxygen atom in the –COOH group gains approximately 1.11 electrons (Table S2), further substantiating the occurrence of this electron transfer process. This electron redistribution at the interface gives rise to a pronounced polarization effect, which enhances the electrostatic attraction between the electrode surface and electrolyte ions, thereby effectively boosting the pseudocapacitive performance. In addition, we calculated the electron localization function (ELF) to further elucidate the electronic behavior in the functionalized regions. As shown in Fig. 3j, the ELF of –COOH-functionalized graphene exhibits high localization in the O–C and C=O bonds, as well as around the oxygen atoms, indicating strong covalent bonding and the presence of lone pair electrons. These findings are consistent with the charge accumulation observed in the differential charge density map, confirming effective electron transfer from the graphene substrate to the –COOH region, predominantly localized around the oxygen-containing groups. By contrast, the ELF map of –OH-functionalized graphene displays more limited high-ELF regions, mainly around the O–H bond and adjacent oxygen atom, which aligns with its relatively weaker influence on the overall electronic structure. Furthermore, we calculated the total density of states (TDOS) to gain deeper insight into the influence of functional groups on the electronic state distribution (Fig. 3k). The results reveal that –COOH-functionalized graphene exhibits a pronounced TDOS peak in the range of 0–0.2 eV, which may originate from localized electronic states introduced by the –COOH groups or hybridized orbitals between the functional groups and the graphene substrate. This indicates the presence of redox-active sites within this energy range, contributing to enhanced pseudocapacitive behavior. In contrast, –OH-functionalized graphene shows relatively minor changes in TDOS, suggesting its limited ability to modulate the electronic structure. Figure 3l presents the adsorption configurations and corresponding adsorption energies of H^+^ ions on both functionalized surfaces. It is clearly observed that H^+^ exhibits a lower adsorption energy on the –COOH-functionalized graphene (–1.13 eV) compared to the –OH-functionalized counterpart (–1.04 eV), indicating that –COOH groups can more stably capture H^+^ ions. This facilitates faster ion insertion and extraction processes at the electrode surface, thereby enhancing the electrochemical response rate and pseudocapacitive performance. In summary, –COOH-functionalized graphene demonstrates superior performance over –OH-functionalized graphene in terms of electronic structure regulation, enhanced electron localization, and H^+^ adsorption capability. These theoretical results are consistent with experimental observations and further validate its potential for application in the design of high-performance pseudocapacitors. To investigate the synergistic effect of –COOH and –OH functional groups, we performed theoretical calculations for a model with a –COOH to –OH ratio of 1:1. The results indicate that the coexistence of both functional groups significantly enhances the charge polarization of the graphene surface (Fig. S5i) and forms stable active sites centered on the oxygen atoms (Fig. S5j), which facilitates the adsorption of electrolyte ions. The TDOS in Fig. S5k reveals a significant increase near the Fermi level, with a distinct peak appearing at 0.2 eV, similar to the effect of a single carboxyl group, collectively creating more redox-active sites. Furthermore, this model exhibits a strong adsorption energy of − 1.15 eV (Fig. S5l), demonstrating a superior ion-trapping capability compared to singly functionalized models and greatly promoting ion transport efficiency at the electrode surface. In summary, the synergistic effect of –COOH and –OH functional groups significantly enhances the material's pseudocapacitive performance by cooperatively optimizing the electronic structure and increasing surface activity.

### Negative Electrode

#### Materials Characterization

**Fig. 4:**
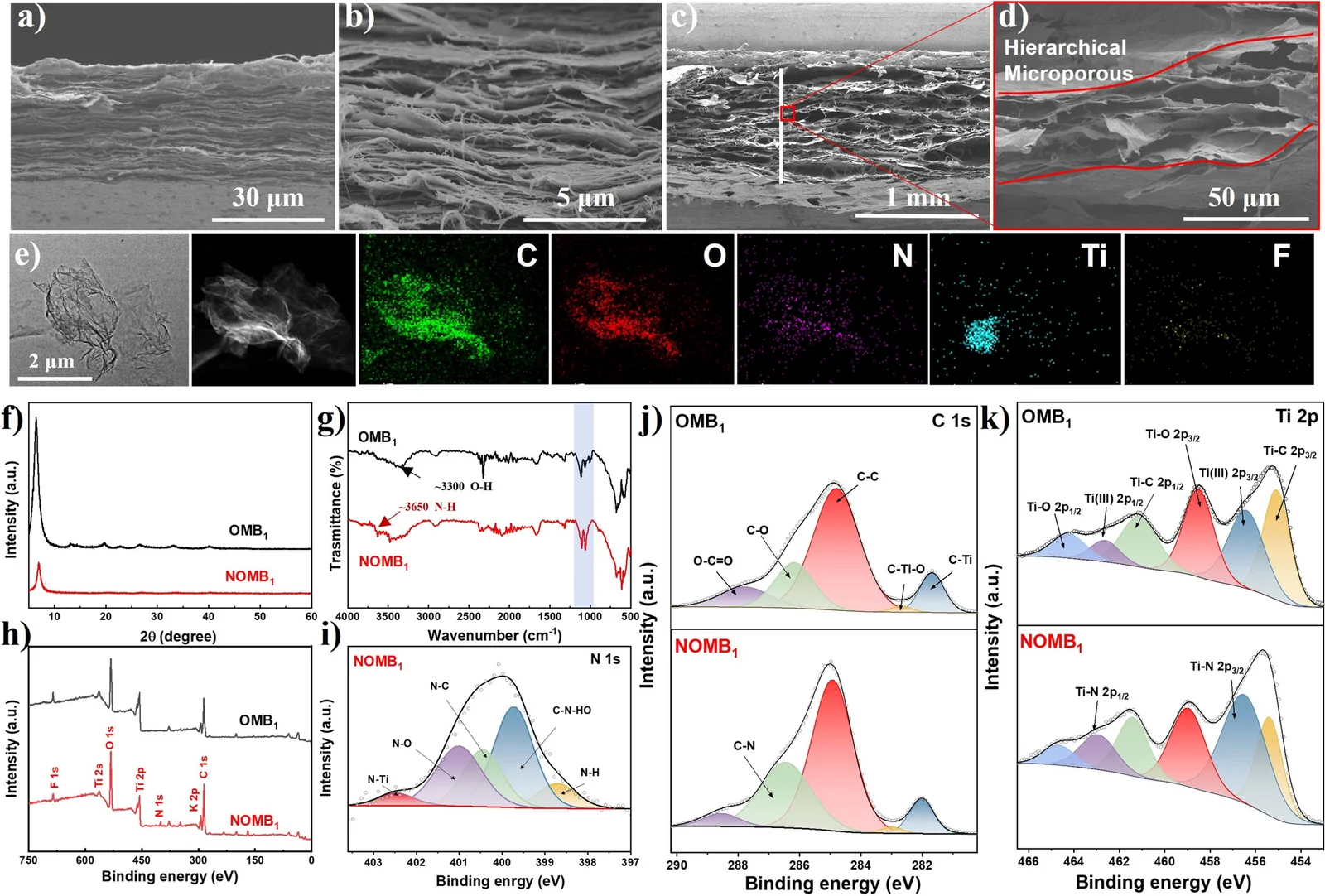
Morphology and chemical characterization of OMB_1_ and NOMB_1_ nanopapers. **a** Cross-sectional SEM image of the OMB_1_ nanopaper; **b** magnified cross-sectional SEM image of OMB_1_; **c** cross-sectional SEM image of the NOMB_1_ nanopaper; **d** magnified cross-sectional SEM image of NOMB_1_;** e** TEM image and corresponding EDS elemental mapping of the NOM; OMB_1_ and NOMB_1_ nanopaper of: **f** XRD spectra; **g** FTIR spectra; **h** XPS survey spectra; **i** high-resolution N 1* s* XPS spectrum of the NOMB_1_ nanopaper; **j** high-resolution C 1*s* XPS spectrum; **k** high-resolution Ti 2*p* XPS spectrum

For the negative electrode, we further developed a gas expansion strategy for MXene nanopapers based on our previous alkali-treated MXene. Specifically, we employed hydrazine, which decomposes at high temperatures to generate gas, enabling the expansion of MXene layers while simultaneously tuning their surface chemistry. Figure 4a–d shows the changes in the nanopaper cross sections before and after hydrazine treatment. Figure 4a, b display the cross-sectional images of nanopapers solely based on KOH-treated MXene (denoted as OMB_1_). The introduction of K^+^ ions induce wrinkling of MXene nanosheets and enlarges the interlayer spacing, resulting in a cross section that is relatively loose rather than densely packed, compared with MXene. In comparison, after gas expansion treatment (Fig. 4c, d), the NOMB_1_ nanopapers exhibit a more favorable spatial architecture. This is attributed to the rapid decomposition of hydrazine under high temperature and pressure, according to the reaction equation: 3N_2_H_4_⋅H_2_O→N_2_ + 4NH_3_ + 3H_2_O, which generates gas that instantaneously expands and overcomes the van der Waals forces between the MXene layers, thus forming a spatial architecture. Further magnified images reveal that the expansion process not only disrupts the van der Waals forces between layers but also within layers, leading to the formation of a hierarchically spatial architecture. Such a hierarchical architecture significantly facilitates fast ion transport by providing low tortuosity ion diffusion pathways. Figure 4e presents the transmission electron microscopy (TEM) image and corresponding energy-dispersive X-ray spectroscopy (EDS) elemental mapping of the NOM sample. The TEM image on the left reveals that the MXene nanosheets exhibit pronounced wrinkles, indicating good flexibility and a well-defined layered structure. The EDS elemental maps on the right show a uniform distribution of elements across the MXene region, suggesting a homogeneous elemental composition. Furthermore, the presence of nitrogen confirms the successful introduction of –NH_2_ functional groups. To investigate the effect of ionic cross-linking density on the hydrazine-induced gas expansion behavior, we characterized the morphology of samples prepared with different KOH contents, as shown in Fig. S6. Figure S6a–c shows the planar and cross-sectional images of nanopapers with KOH:MXene mass ratios of 0:1, 1:1, and 2:1, respectively. From the planar images (Fig. S6a_1_–c_1_), it can be observed that the degree of wrinkling increases with higher KOH content, which facilitates electrolyte infiltration. The cross-sectional images (Fig. S6a_2_–c_2_) show that the nanopaper thickness also increases with increasing KOH content, attributed to enhanced wrinkling caused by stronger ionic cross-linking. After hydrazine treatment, the planar images (Fig. S6d_1_–f_1_) similarly demonstrate greater wrinkling with increasing KOH content. Meanwhile, the cross-sectional images (Fig. S6d_2_–f_2_) reveal that the pore size and nanopaper thickness both increase with higher KOH content. The specific surface area measurements (Fig. S6g–i) reveal that NOMB exhibits a larger surface area than OMB after hydrazine-assisted gas expansion, indicating that the gas expansion process plays a crucial role in constructing porous structures and enlarging the surface area, thereby facilitating ion transport and enhancing the exposure of electrochemically active sites.

To gain deeper insight into the changes in surface functional groups and structural characteristics of the nanopapers, comprehensive chemical characterizations were conducted. Figure 4f presents the XRD spectra of the OMB_1_ and NOMB_1_ nanopapers. The (002) diffraction peak intensity of the NOMB_1_ sample is significantly reduced, which is primarily attributed to signal attenuation caused by the increased nanopaper thickness. Figure 4g shows the FTIR spectra of the OMB_1_ and NOMB_1_ nanopapers. In OMB_1_, the –OH stretching vibration appears at 3300 cm^−1^. In NOMB_1_, this peak undergoes a noticeable redshift, and a new N–H stretching vibration emerges around 3650 cm^−1^. In the 1200–1000 cm^−1^ region, the stretching vibrations of C–N single bonds are present, but their relatively weak signals are partially obscured by overlapping C–O absorption peaks from the BC component [42]. These observations indicate the introduction of –NH_2_ groups, likely arising from the adsorption of polar molecules on the MXene surface or the formation of new hydrogen bonds. Figure 4h displays the XPS survey spectra, where a distinct N 1*s* peak is observed in the NOMB_1_ nanopaper. By calculating the atomic ratios before and after hydrazine treatment, it was found that the C/F ratio increased from 11.14 to 16.98, and the O/F ratio increased from 6.78 to 9.38. This indicates a substantial reduction in –F functional groups, which is favorable for enhancing electrochemical energy storage performance. High-resolution spectra for N 1*s*, C 1*s*, and Ti 2*p* were further deconvoluted. As shown in Fig. 4i, the N 1*s* spectrum of the NOMB_1_ nanopaper confirms the presence of N–Ti, N–C, and N–O bonds. In the C 1*s* spectrum (Fig. 4j), the presence of a C–N peak is also observed. Moreover, the binding energies of Ti(III) 2*p*_1/2_ and 2*p*_3/2_ shift positively, indicating that the introduction of nitrogen enhances the local electronegativity, leading to chemical shifts (Fig. 4k). To investigate the influence of the KOH:MXene mass ratio on the high-temperature reaction with hydrazine, structural features of nanopapers with different degrees of ionic cross-linking were further analyzed. As shown in Fig. S6j, the FTIR spectra of OMB_0_, OMB_1_, and OMB_2_ demonstrate that the intensity of the –OH stretching peak increases with higher KOH content, indicating a greater amount of –OH functional groups on the nanopaper surface. In the XRD spectra (Fig. S6k), the (002) peak shifts to lower angles as KOH content increases, suggesting that the incorporation of K^+^ ions effectively enlarge the MXene interlayer spacing consistent with our previous findings. Additionally, as the KOH amount increases, the –NH_2_ absorption peaks in the FTIR spectra of NOMB nanopapers become more pronounced, while the XRD peak intensity gradually decreases due to the increase in nanopaper thickness (Fig. S6l, m).

#### Electrochemical Performance

**Fig. 5:**
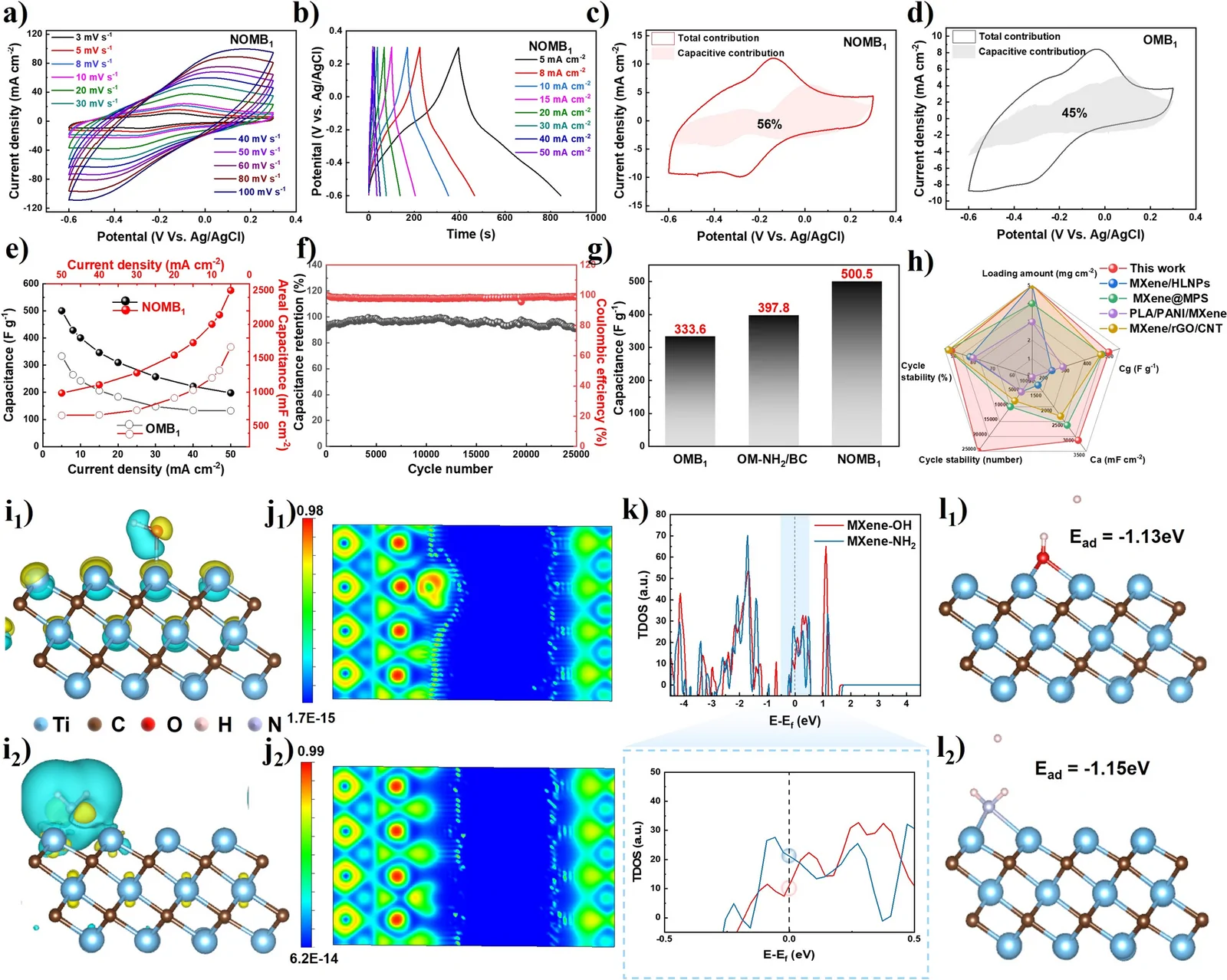
Electrochemical performance of OMB_1_ and NOMB_1_ nanopapers. **a** CV curves of the NOMB_1_ nanopaper at different scan rates; **b** GCD curves of the NOMB_1_ nanopaper at various current densities; **c** CV curve of the NOMB_1_ nanopaper at 3 mV s^−1^, with the shaded area indicating the surface capacitive contribution; **d** CV curve of the OMB_1_ nanopaper at 3 mV s^−1^, with the shaded area indicating the surface capacitive contribution; **e** comparison of specific capacitance between OMB_1_ and NOMB_1_ composite nanopapers; **f** cycling stability and corresponding coulombic efficiency of the NOMB_1_ nanopaper at 20 mA cm^−2^; **g** comparison of specific capacitance between OMB_1_, OM–NH_2_/BC and NOMB_1_ at current densities ranging from 5 to 50 mA cm^−2^; **h** radar charts show the excellence of NOMB_1_ porous nanopapers in all aspects of performance; side views of differential charge density: **i**_**1**_ MXene–OH, **i**_**2**_ MXene–NH_2_; electron localization function (ELF) plots: **j**_**1**_ MXene–OH, **j**_**2**_ MXene–NH_2_; **k** DOS at the Fermi level between MXene–OH and MXene–NH_2_; optimized structures of H^+^ adsorption on MXene–OH (**l**_**1**_) and MXene–NH_2_ (**l**_**2**_), along with their corresponding adsorption energies

The microstructure and chemical composition have a significant impact on the electrochemical performance of electrode materials. Figures 5a, b and S7a, b present the CV and GCD curves of the OMB_1_ and NOMB_1_ nanopapers, respectively. Compared with the OMB_1_ nanopaper, the NOMB_1_ nanopaper exhibits a larger CV curve area and longer charge–discharge duration in the GCD curves, indicating superior energy storage capability. Combined with the chemical characterization results, it is evident that the NOMB_1_ nanopaper successfully introduces new surface functional groups (–NH_2_), which effectively increases the number of pseudocapacitive active sites. Using the same pseudocapacitive fitting method as previously applied to the graphene electrodes, we analyzed the capacitive contributions of the OMB_1_ and NOMB_1_ nanopapers. As shown in Fig. 5c, d, the NOMB_1_ nanopaper exhibits a significantly higher pseudocapacitive contribution than the OMB_1_ nanopaper, confirming the effectiveness of our surface chemistry regulation strategy for MXene. Figure 5e compares the specific capacitance of the two nanopapers, at a current density of 5 mA cm^−2^, the specific capacitance of the NOMB_1_ nanopaper reaches 500.5 F g^−1^ (2502.5 mF cm^−2^), which is 150% of the OMB_1_ nanopaper. In addition, the NOMB_1_ nanopaper also demonstrates excellent cycling stability, retaining 91.35% of its initial capacitance after 25,000 charge–discharge cycles (Fig. 5f). In comparison, OMB1 retains 83.5% of its capacitance after 10,000 cycles (Fig. S7c), further highlighting the superiority of its pore structure. EIS analysis was conducted to further investigate the electrochemical behavior of the electrodes. Figure S7d shows the Nyquist plots of the two electrodes. It is evident from the figure that after surface modification with amino groups (–NH_2_), the R_ct_ of the electrode decreases from 0.87 to 0.54 Ω, indicating a smoother charge transfer process. Figure S7e presents the plot of real impedance (Z') versus the inverse square root of frequency (ω^−1/2^), along with linear fitting. The slope of the fitted curve is closely related to the ion diffusion coefficient; a smaller slope suggests a higher diffusion coefficient, implying easier ion diffusion. From the graph, it is clear that the NOMB_1_ electrode exhibits a smaller slope, indicating a higher ion diffusion coefficient and lower diffusion resistance. Figure S7f displays the Bode plots of the two nanopapers. The characteristic time constants are derived from the frequencies corresponding to the peak phase angles. The results show that the time constant of the OMB_1_ nanopaper is approximately 68.22 s, while that of the NOMB_1_ nanopaper is about 38.31 s, indicating that the latter possesses faster electrochemical kinetics and more rapid charge response. To further elucidate the relationship between reduced Rct and improved electrochemical performance, we measured the electrical conductivity of OM and NOM under the premise of excluding structural differences and focusing solely on the influence of surface functional groups. Figure S7g presents the conductivity test results. The conductivity of the functionalized NOM is approximately 440% higher than OM. This significant enhancement clearly demonstrates that after hydrothermal treatment with hydrazine not only optimizes the charge transport pathways but also improves the overall electronic conductivity—further explaining the origin of its superior electrochemical performance. Meanwhile, –NH_2_ groups can replace terminal functional groups (such as –F) on the surface of MXene under alkaline conditions, thereby modulating its surface chemical properties. To verify the effectiveness of surface amination on MXene, the MXene subjected to hydrazine hydrothermal treatment was ground and then vacuum-filtered with BC to prepare OM–NH_2_/BC nanopaper, followed by electrochemical performance testing. As shown in the CV and GCD curves in Fig. S8a, b, the OM–NH_2_/BC nanopaper exhibited clear redox peaks were observed, at low scan rates, indicating pronounced pseudocapacitive behavior. Further comparison of the CV curves at the same scan rate showed that the OM–NH_2_/BC exhibited a markedly larger integrated area and a stronger peak intensity than the OMB nanopaper (Fig. S8c). In addition, specific capacitances of MOB_1_, OM–NH_2_/BC, and NOMB_1_ nanopapers were compared at scan rates of 5 mA cm^−2^ (Fig. 5g). These results indicate that the introduction of –NH_2_ groups effectively enhances pseudocapacitive charge storage behavior. As shown in Fig. 5h, the NOMB_1_ nanopaper exhibits outstanding performance in terms of specific capacitance and cycling stability compared to other MXene-based electrode materials, highlighting its remarkable competitive advantage.

To systematically investigate the influence of alkali-treated MXene degree and the extent of functional group substitution on electrochemical performance, we conducted hydrothermal treatment of MXene nanopapers with varying KOH content using hydrazine, resulting in OMB_0_, OMB_1_, and OMB_2_ nanopapers and their corresponding treated samples, NOMB_0_, NOMB_1_, and NOMB_2_. Figures 5 and S8, S9 present the CV curves, GCD curves, and specific capacitance comparisons of the nanopapers before and after treatment. As shown, all hydrazine-treated electrodes exhibit distinct redox peaks even at high scan rates, confirming that the introduction of amino groups effectively enhances pseudocapacitive behavior. As the KOH content increases, the specific capacitance of the untreated OMB nanopapers increases accordingly. At a current density of 5 mA cm^−2^, the specific capacitances are: 238.9 F g^−1^ (1194.6 mF cm^−2^) for OMB_0_, 333.6 F g^−1^ (1668.0 mF cm^−2^) for OMB_1_, and 281.2 F g^−1^ (1406.0 mF cm^−2^) for OMB_2_. This suggests that the introduction of –OH facilitate interlayer spacing expansion within the MXene structure, thereby improving the capacitive performance. Following hydrazine treatment, the specific capacitance of the samples first increases and then slightly decreases: 296.6 F g^−1^ (1482.9 mF cm^−2^) for NOMB_0_, 500.5 F g^−1^ (2502.5 mF cm^−2^) for NOMB_1_, and 484.5 F g^−1^ (2422.5 mF cm^−2^) for NOMB_2_. The initial performance enhancement is primarily attributed to the more effective substitution of terminal groups (e.g., –F) with –NH_2_ under alkaline conditions, as well as the formation of porous structures within the nanopaper, which increases the number of pseudocapacitive active sites. However, under excessively strong alkaline conditions, an excess of –OH may excessively occupy surface active sites, thereby hindering the subsequent incorporation of –NH_2_.

#### Theoretical Calculation

Based on the experimental results above, we further employed DFT calculations to gain deeper insight into the contribution of surface functional groups on MXene to its electrochemical performance and their effect on H^+^ adsorption behavior in the electrolyte. To simplify the model, we constructed MXene surfaces functionalized with a single –OH or –NH_2_ group. The optimized structures and their corresponding differential charge density maps are shown in Fig. 5i. In the –OH-functionalized model (Fig. 5i1), electron accumulation is mainly concentrated around the O atom, suggesting that the highly electronegative O atom effectively attracts electrons and serves as an active site for proton adsorption. Conversely, electron depletion is primarily distributed at the Ti–C interface, indicating electron donation from Ti to the –OH group. The differential charge density of the –NH_2_ functionalized surface (Fig. 5i2) shows a broader electron depletion region spanning the substrate, indicating that the amino group promotes electron delocalization across the MXene surface. The ELF map in Fig. 5j1 shows a highly localized region (ELF = 0.98) corresponding to the polar O–H bond, suggesting strong covalent bonding that facilitates stable H^+^ adsorption. The ELF map in Fig. 5j2 further reveals electron distribution extending from the N atom to the Ti–N bond, exhibiting pronounced delocalization characteristics. This indicates that nitrogen doping facilitates the formation of an electron–sharing network. Such dynamic Ti–N–H^+^ interactions promote rapid H^+^ adsorption/desorption, thereby enhancing pseudocapacitive behavior. Moreover, the delocalized electronic network helps reduce interfacial resistance, as confirmed by the TDOS plot in Fig. 5k. Compared to –OH, the –NH_2_ functional group contributes a higher density of electronic states near the Fermi level (E − E_f_ = 0 eV), indicating superior electronic conductivity. We also calculated the H^+^ adsorption energies for both functional groups in a sulfuric acid electrolyte (Fig. 5i). The results indicate that H^+^ adsorption on the MXene–OH surface involves a structural reconstruction, where the oxygen atom in the –OH group transitions from monodentate coordination (Ti–O) to a tridentate μ_3_–O configuration (coordinated with three Ti atoms). FTIR was conducted on the OMB nanopapers before and after H^+^ adsorption/desorption, and the results (Fig. S10) confirm the presence of μ_3_–O structures in the post–test nanopapers. Specifically, the O–H bending vibration peak near 1600 cm^−1^ splits, indicating different hydrogen bonding environments, and new sharp peaks emerge in the 500–800 cm^−1^ range, corresponding to the vibrational modes of μ_3_–O bridging. After subtracting the energy cost of structural reconstruction, the final H^+^ adsorption energy is calculated to be –1.13 eV. Under acidic conditions, the deprotonation of –OH leads to the formation of negatively charged –O^−^ species. To stabilize this charge, the oxygen atom migrates into a triangular site formed by three adjacent Ti atoms, creating a μ_3_–O bridge. This configuration is particularly stable in acidic environments and represents a key step in the transition from –OH to oxygen termination. Although the difference in H^+^ adsorption energy between –OH and –NH_2_ is relatively small (ΔΔE = 0.02 eV), the superior electron delocalization and dynamic bonding behavior of –NH_2_ make it more favorable than –OH in enhancing overall electrochemical performance.

### ASC Electrochemical Performance

**Fig. 6:**
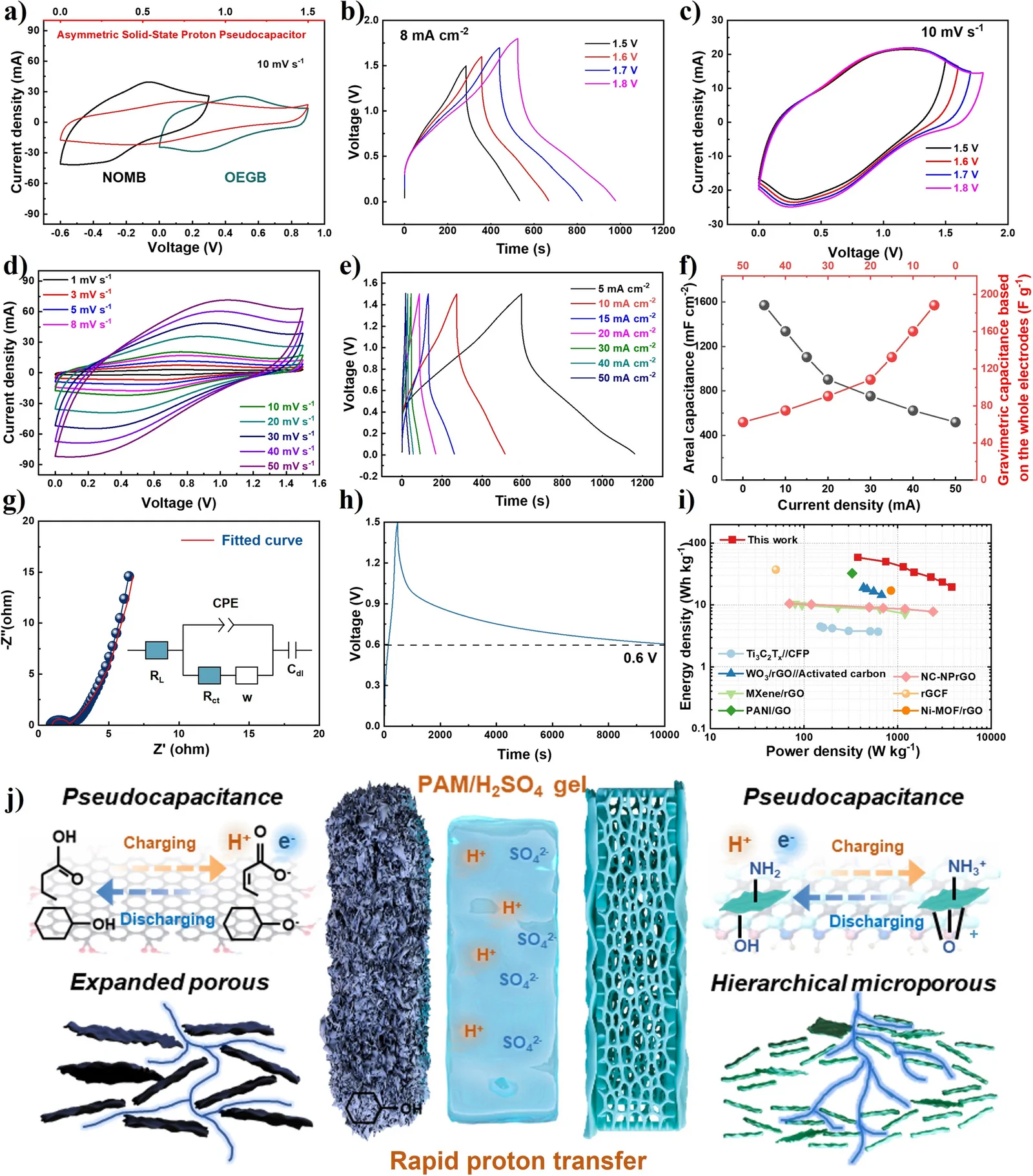
Electrochemical performance of the solid-state asymmetric supercapacitor (ASC) with NOMB as the positive electrode and OEGB as the negative electrode. **a** CV curves of NOMB, OEGB, and the assembled asymmetric device at a scan rate of 10 mV s^−1^; **b** GCD curves of the assembled device at a current density of 8 mA cm^−2^ within a voltage window of 1.5–1.8 V; **c** CV curves at different voltage windows at a scan rate of 10 mV s^−1^; **d** CV curves at various scan rates within the 0–1.6 V voltage range; **e** GCD curves at different current densities within the 0–1.6 V voltage range; **f** areal and gravimetric capacitances of the ASC at current densities ranging from 5 to 50 mA cm^−2^; **g** Nyquist plot of the asymmetric supercapacitor with an inset showing the equivalent circuit model; **h** self-discharge curve of ASC; **i** Ragone plot comparing the performance of the ASC with existing MXene-based and graphene-based supercapacitors; **j** the energy storage mechanism of ASC

Given that both the cathode material NOMB and the anode material OEGB exhibit excellent electrochemical performance, a solid-state asymmetric supercapacitor (ASC) was constructed using NOMB as the positive electrode and OEGB as the negative electrode, with a device total active material loading of 10 mg cm^−2^. As shown in Fig. 6a, the assembled ASC achieves a maximum operating voltage of 1.5 V, significantly broadening the voltage window and laying a foundation for achieving high energy density. Figure 6b, c presents the CV and GCD curves under different voltage ranges (1.5–1.8 V). As the voltage window increases, the shape of the curves remains consistent without noticeable distortion or signs of electrolyte decomposition, indicating stable and reversible electrochemical behavior within a wide operating voltage range. To further evaluate the rate capability, the device was tested at various scan rates and current densities (Fig. 6d, e). At a current density of 5 mA cm⁻^2^, the ASC delivers a high specific capacitance of 188.4 F g^−1^, corresponding to an area capacitance of 1570 mF cm^−2^, demonstrating excellent energy storage capability (Fig. 6f). EIS measurements were performed to further assess the compatibility between NOMB and OEGB electrodes (Fig. 6g). The Nyquist plot reveals low R_s_ and R_ct_, suggesting good interfacial conductivity and efficient charge transport processes, which enable high-power output under large current conditions. Additionally, the ASC maintains a voltage of approximately 0.6 V after open-circuit standing for 10,000 s, exhibiting excellent self-discharge suppression, which is beneficial for long-term energy storage and device reliability (Fig. 6h). In terms of comprehensive performance, the NOMB//OEGB ASC developed in this work was compared with representative MXene-based and graphene-based supercapacitors reported in the literature (Fig. 6i) [14, 44–49]. The results demonstrate that the device achieves a high energy density of 58.9 Wh kg^−1^ and a power density of 3802 W kg^−1^, significantly outperforming most previously reported asymmetric supercapacitors (Tables S3-S5), thereby exhibiting strong potential for practical applications. The energy storage mechanism is illustrated in Fig. 6j, highlighting the high degree of synergy between the structural engineering and interfacial design of the negative and positive electrode materials. In the OEGB negative electrode, expanded-oxidation graphene fragments rich in –COOH groups form a stable porous network, which enhances the specific surface area of the electrode and provides abundant ion diffusion channels. In the NOMB positive electrode, the gas expansion effect induced by hydrazine treatment promotes the formation of hierarchical micropores between MXene layers. This effectively mitigates restacking, increases the exposure of electrochemically active sites, and improves electrolyte wettability. The coordinated design of surface functional groups (–NH_2_ and –COOH) and pore structure in both electrodes enables well-matched electronic structure modulation, interfacial compatibility, and charge storage mechanisms. As a result, a high-performance solid-state asymmetric proton-type pseudocapacitor with high energy density, high-power output, and rapid response has been successfully achieved.

**Fig. 7:**
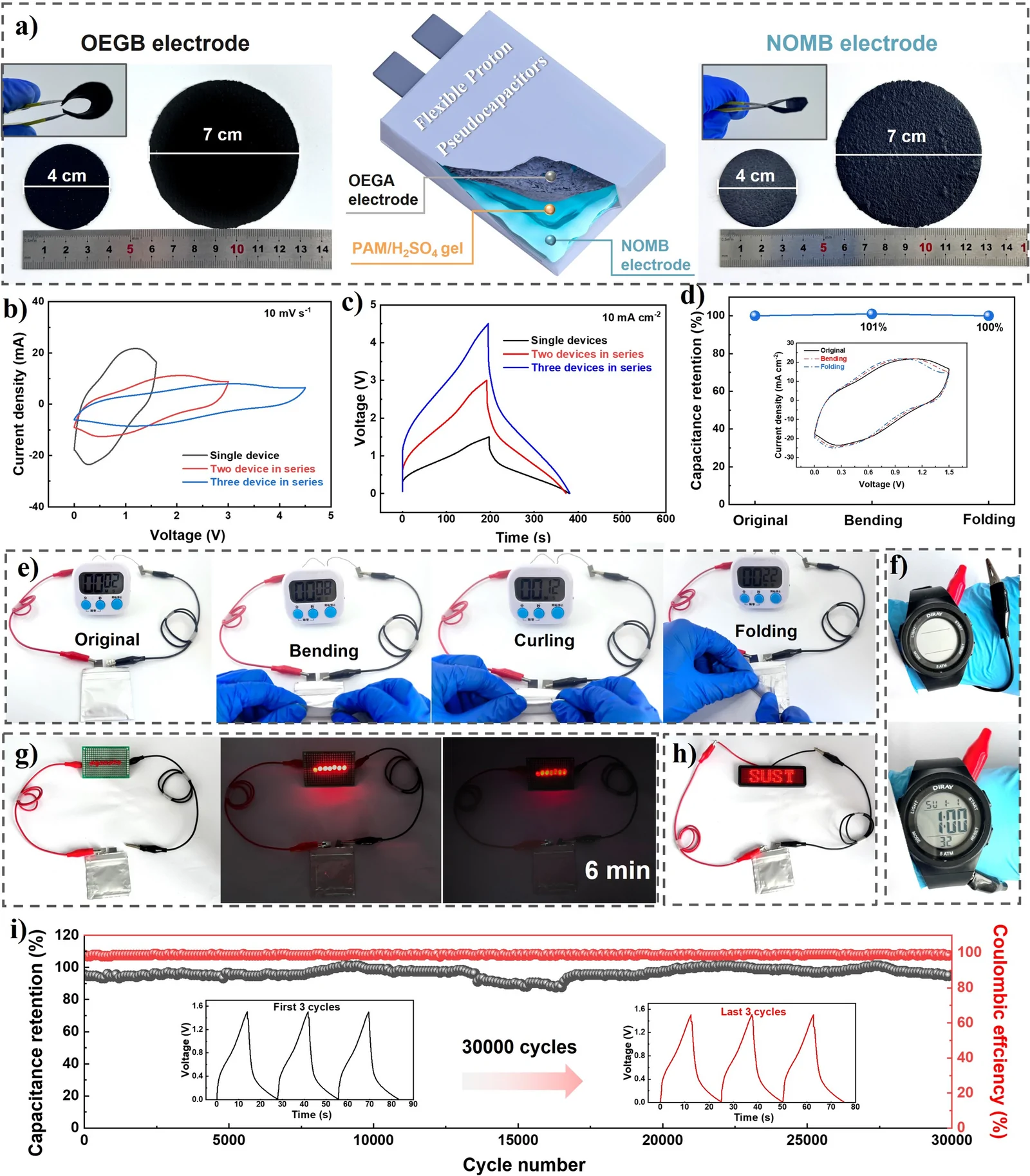
**a** Structure of the NOMB//OEGB ASC device and demonstration of the flexibility of both positive and negative electrodes; **b** CV and **c** GCD curves of one, two, and three ASC devices connected in series; **d** CV curves of the NOMB//OEGB ASC under different bending and folding states at a scan rate of 10 mV s^−1^; **e** optical images of the ASC device powering a digital timer under various deformation states (bending, folding, curling); **f** application of the ASC device as a wearable energy storage strap for powering a display; **g** power seven LED lights continuously for an extended period; **h** power a display panel showing the word “SUST”; **i** cycling stability and corresponding coulombic efficiency of the NOMB//OEGB device at a current density of 50 mA cm^−2^. The inset shows the GCD curves of the first and the last three cycles

To evaluate the practical applicability of the NOMB//OEGB ASC developed in this work, a series of application-oriented tests were conducted. As shown in Fig. 7a, the ASC device consists of a positively and negatively charged electrode, both of which can be fabricated on a large scale and exhibit good flexibility, thus contributing significantly to the overall flexibility of the device. Figure 7b, c presents the CV curves of a single ASC, as well as two and three devices connected in series at a scan rate of 10 mV s^−1^, and the GCD curves at a current density of 10 mA cm^−2^. The results indicate that the device possesses excellent series compatibility and favorable electrochemical performance. Furthermore, the mechanical stability of the device under various deformation conditions was evaluated. As shown in Fig. 7d, the capacitance performance remains nearly unchanged under bending and folding states, indicating outstanding flexibility and structural robustness. Owing to its exceptional mechanical flexibility, the ASC device can stably power a timer under various deformations, such as bending, curling, and folding (Fig. 7e), which fully validates its feasibility for practical applications. More importantly, after bending at different angles and undergoing 1000 cycles, the device retains 87% of its initial capacitance, demonstrating its outstanding mechanical stability (Fig. S11e). The results indicate that the device maintains stable electrochemical performance under repeated mechanical stress, further confirming its reliability for flexible applications. Figure 7f further highlights the device’s high reliability and outstanding adaptability for use in flexible and wearable electronic devices. In addition, the device can continuously drive seven LED lights for an extended period and successfully illuminate an LED pattern displaying the word “SUST” (as shown in Fig. 7g, h), further demonstrating its potential in practical lighting and display applications. As shown in Fig. 7i, the device exhibits excellent stability over 30,000 charge–discharge cycles, maintaining 89.7% capacitance retention and 98.9% coulombic efficiency relative to the initial values. Meanwhile, we compared the surface SEM images before and after cycling, and the results show no cracks or pores on the surface after cycling, indicating that our material exhibits excellent stability (Fig. S11a–d). This outstanding cycling performance significantly surpasses that of previously reported graphene- and MXene-based supercapacitors (Table S5). The superior electrochemical stability can be mainly attributed to the ultrafine 3D network structure constructed by BC. This unique 3D framework not only provides excellent mechanical support and flexibility to accommodate volume changes during repeated charge–discharge cycles, thereby effectively mitigating structural degradation and electrode pulverization, but also promotes the formation of interconnected porous channels that facilitate rapid ion diffusion and electrolyte penetration. Moreover, the abundant hydroxyl groups on BC’s surface serve as active sites for strong interfacial bonding with the electrode materials, ensuring stable integration and efficient electron transport. Additionally, BC’s hydrophilic nature enhances electrolyte wettability, further contributing to sustained electrochemical activity and prolonged cycle life. Collectively, these factors synergistically enable the device to maintain outstanding cycling stability over extended operation. In summary, the NOMB//OEGB ASC demonstrates outstanding electrochemical performance, excellent flexibility, and high operational stability, making it a promising candidate as a reliable and high-performance energy storage device for next-generation flexible and wearable electronic applications.

## Conclusion

In conclusion, we have developed an advanced asymmetric solid-state proton-type pseudocapacitor by integrating modified MXene and graphene electrodes through gas-driven expansion and surface chemistry optimization. The rapid evaporation and mixed acid oxidation of graphene significantly improved its electrochemical performance, with a 1:1 ratio of –COOH and –OH functional groups delivering the highest pseudocapacitive contribution. DFT simulations revealed that –COOH groups enhance electron transfer and proton adsorption. Similarly, MXene modified with KOH and hydrazine-assisted hydrothermal reactions boosted electron delocalization and proton adsorption. The assembled device demonstrated an energy density of 58.9 Wh kg^−1^ and a power density of 3802 W kg^−1^ at a mass loading of 10 mg cm^−2^, retaining 89.7% of its capacitance after 30,000 cycles. Furthermore, the device demonstrated excellent mechanical flexibility, capable of withstanding various deformations, making it suitable for flexible and wearable energy storage applications. These results underscore the potential of 2D thick electrodes for high-performance, flexible energy storage systems.

## Supplementary Information

Below is the link to the electronic supplementary material.

Supplementary file1 (DOCX 21655 KB)
