# Supplementary material for: Synergistic Design of Flexible Nanopapers for High-Performance Proton Pseudocapacitors
Source: Nanomicro Lett. 2026 Jan 5;18:158. doi: 10.1007/s40820-025-01989-6 (PMC12765798; doi:10.1007/s40820-025-01989-6)
Supplement: Supplementary file 1 — Supplementary file1 (DOCX 21655 KB) [file 40820_2025_1989_MOESM1_ESM.docx]

Supporting Information for

**Synergistic Design of Flexible Nanopapers for High-Performance Proton Pseudocapacitors**

Jiayue Dong^1^, Zhaoqing Lu^1,^ *, Li Hua^1, 2,^ *, Zizhan Guo^1^, Xiaoxu Xu^1^, Jinlong Wu^1^, Fengfeng Jia^1^ and Yuanming Wang^1,^ *

^1^College of Bioresources Chemical and Materials Engineering, Shaanxi Provincial Key Laboratory of Papermaking Technology and Specialty Paper Development, National Demonstration Center for Experimental Light Chemistry Engineering Education, Shaanxi University of Science and Technology, Xi’an 710021, P. R. China

^2^Shaanxi ParaDe Advanced Material Technology Co., LTD, Building 5, Western Life Science Park, Fengdong New District, Xixian New Area, Xianyang 712000, P. R. China

*Corresponding authors. E-mail: [luzhaoqing302@163.com](mailto:luzhaoqing302@163.com) (Zhaoqing Lu); [tuliphua@126.com](mailto:tuliphua@126.com) (Li Hua); [yminghit@sust.edu.cn](mailto:yminghit@sust.edu.cn) (Yuanming Wang)

**S1 Calculation Methods**

**S1.1 Electrochemical Calculation Methods**

The gravimetric capacitance and area ratio capacitance of single electrodes was calculated from the discharge portion of galvanostatic charge-discharge (GCD) according to the equations (S1) and (S2), respectively.

$$\begin{aligned} C_{g}=\frac{I\Delta t}{m\Delta V}\#\left( S1 \right) \end{aligned}$$

$$\begin{aligned} C_{a}=\frac{I\Delta t}{S\Delta V}\#\left( S2 \right) \end{aligned}$$

where $C_{g}$ is the gravimetric capacitance (F g^-1^), I is the current density (mA cm-2), $\Delta$t is the discharge time (s), m is the mass of the active substance (mg), $\Delta$V is the voltage window (V), $C_{a}$ is area ratio capacitance (mF cm^-2^), and S is the geometric area of the electrode (cm^-2^), respectively.

The energy density (E) and power density (P) of asymmetric device were calculated

according to the equations (S3) and (S4), respectively.

$$\begin{aligned} E=\frac{1}{2}{CV}^{2}\#\left( S3 \right) \end{aligned}$$

$$\begin{aligned} P=\frac{E\times3600}{\Delta t}\#\left( S4 \right) \end{aligned}$$

**S1.2 DFT Calculation Parameters and Optimized Models**

Density functional theory (DFT) calculations were performed using the Vienna Ab initio Simulation Package (VASP). The exchange–correlation interactions were described using the generalized gradient approximation (GGA) in the form of the Perdew–Burke–Ernzerhof (PBE) functional. A plane-wave cutoff energy of 500 eV was employed. Brillouin zone sampling was carried out using a Monkhorst–Pack k-point mesh. The Gaussian smearing method was applied for electronic state occupation with ISMEAR = 0 and SIGMA = 0.05, which is appropriate for non-metallic systems. Electronic self-consistent calculations were performed using the Fast algorithm (ALGO = Fast), with a convergence criterion of 1 × 10^-5^ eV. For ionic relaxation, a force convergence threshold of 0.02 eV/Å was used (EDIFFG = –0.02), and a maximum of 300 ionic steps (NSW = 300) was allowed. The conjugate gradient algorithm was used for ion relaxation (IBRION = 2), and atomic positions were optimized (ISIF = 2). Spin polarization was considered (ISPIN = 2), and van der Waals interactions were corrected using the DFT-D3 method (IVDW = 12). Symmetry constraints were turned off during the relaxation process (ISYM = 0) to avoid interference with the relaxation path. Real-space projection was set to automatic (LREAL = Auto) to enhance computational efficiency. The structural optimization was performed until full convergence of the energy and geometry was achieved. Wavefunction and electrostatic potential outputs were disabled (LWAVE = .FALSE., LVHAR = .FALSE.) to reduce storage requirements.

**S2 Supplementary Figures and Tables**


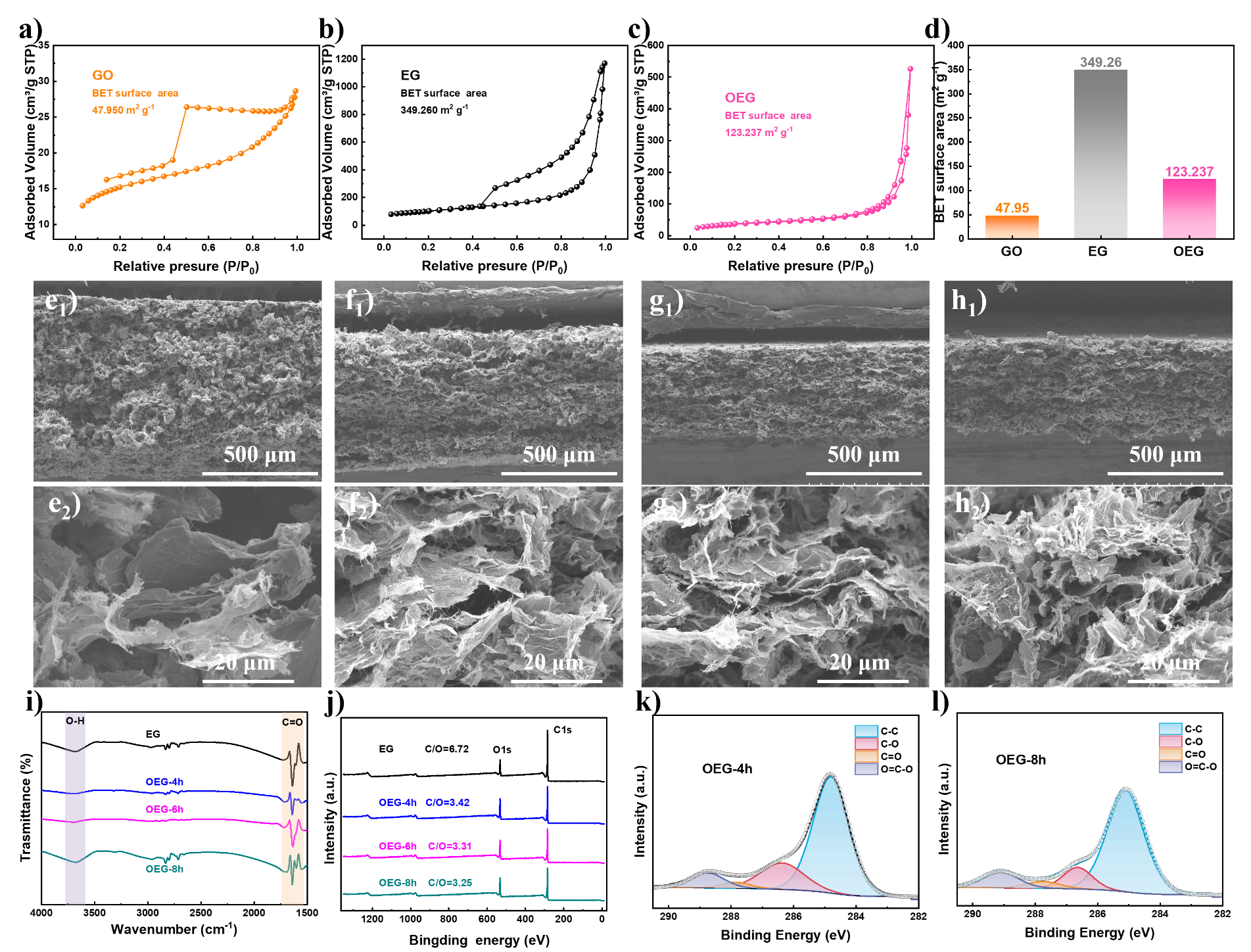


**Fig. S1** Nitrogen adsorption–desorption isotherms **a** GO; **b** EG; **c** OEG; **d** Comparison of specific surface areas of different samples; Cross-sectional morphology and chemical characterization of EGB and OEGB nanopapers with different oxidation durations. Cross-sectional SEM image of **e** EGB nanopaper, **f** OEGB-4h nanopaper, **g** OEGB-6h nanopaper, **h** OEGB-8h nanopaper; The EG and OEG at different oxidation times of **i** FTIR spectra, **j** XPS survey spectra; **k** High-resolution C1s XPS spectrum of OEG-4h; **l** High-resolution C1s XPS spectrum of OEG-8h


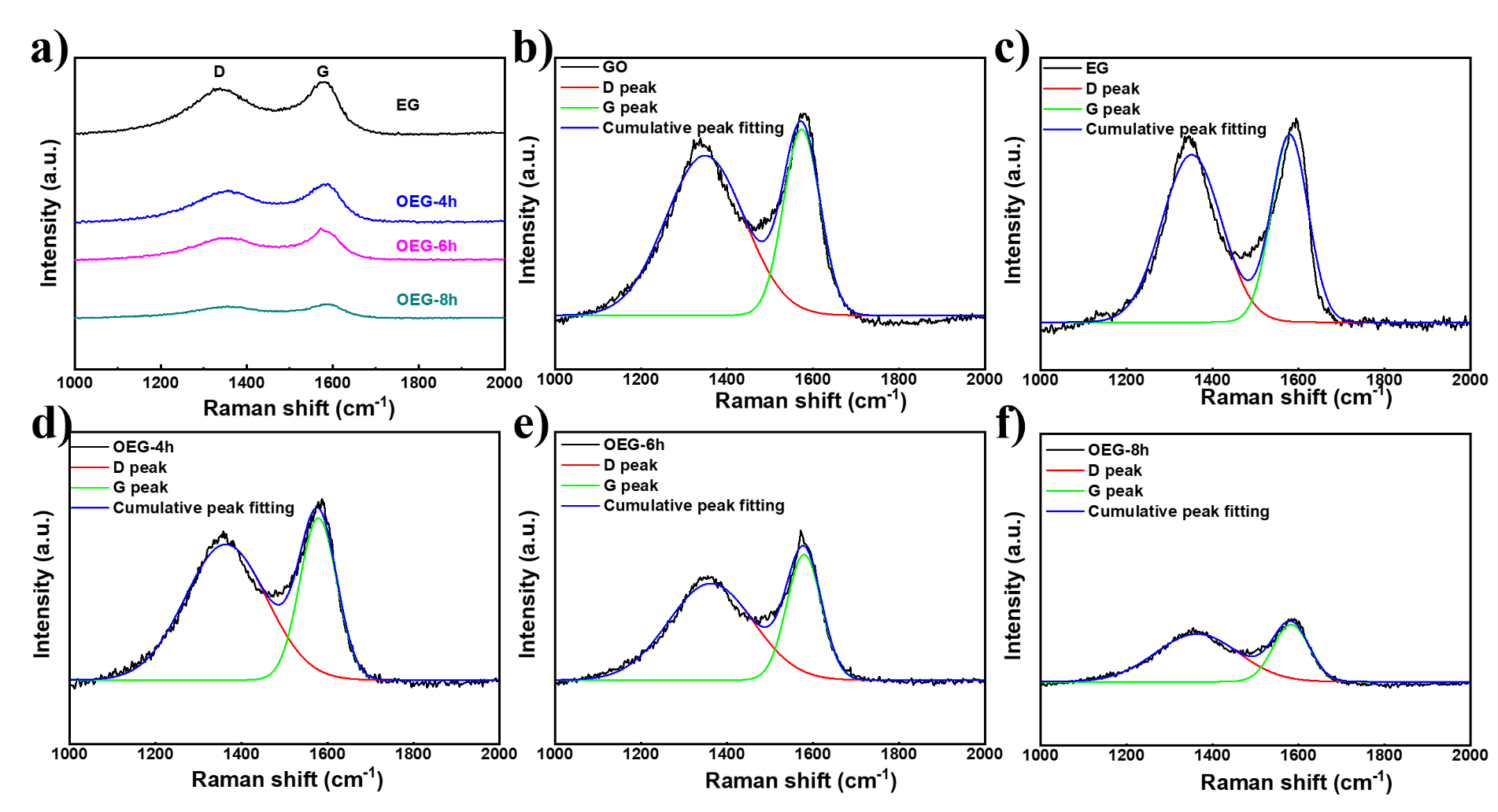


**Fig. S2** **a** Raman spectra of OEG powders with different oxidation durations; Peak fitting of Raman spectra for GO, EG, and OEG powders at various oxidation times: **b** GO powder, **c** EG powder, **d** OEG-4h powder, **e** OEG-6h powder, **f** OEG-8h powder

|  | Integral areas of D peak (I_D_) | Integral areas of G peak (I_G_) | I_d_/I_g_ |
| --- | --- | --- | --- |
| GO | 35295.83 | 19092.34 | 1.848691 |
| EG | 32464.03 | 22424.02 | 1.447735 |
| OEG-4h | 25797.13 | 14076.06 | 1.832696 |
| OEG-6h | 19119.1 | 10209.02 | 1.872766 |
| OEG-8h | 9223.203 | 4839.371 | 1.905868 |

**Table S1** Peak fitting results of Raman spectra for GO, EG, and OEG powders with different oxidation durations


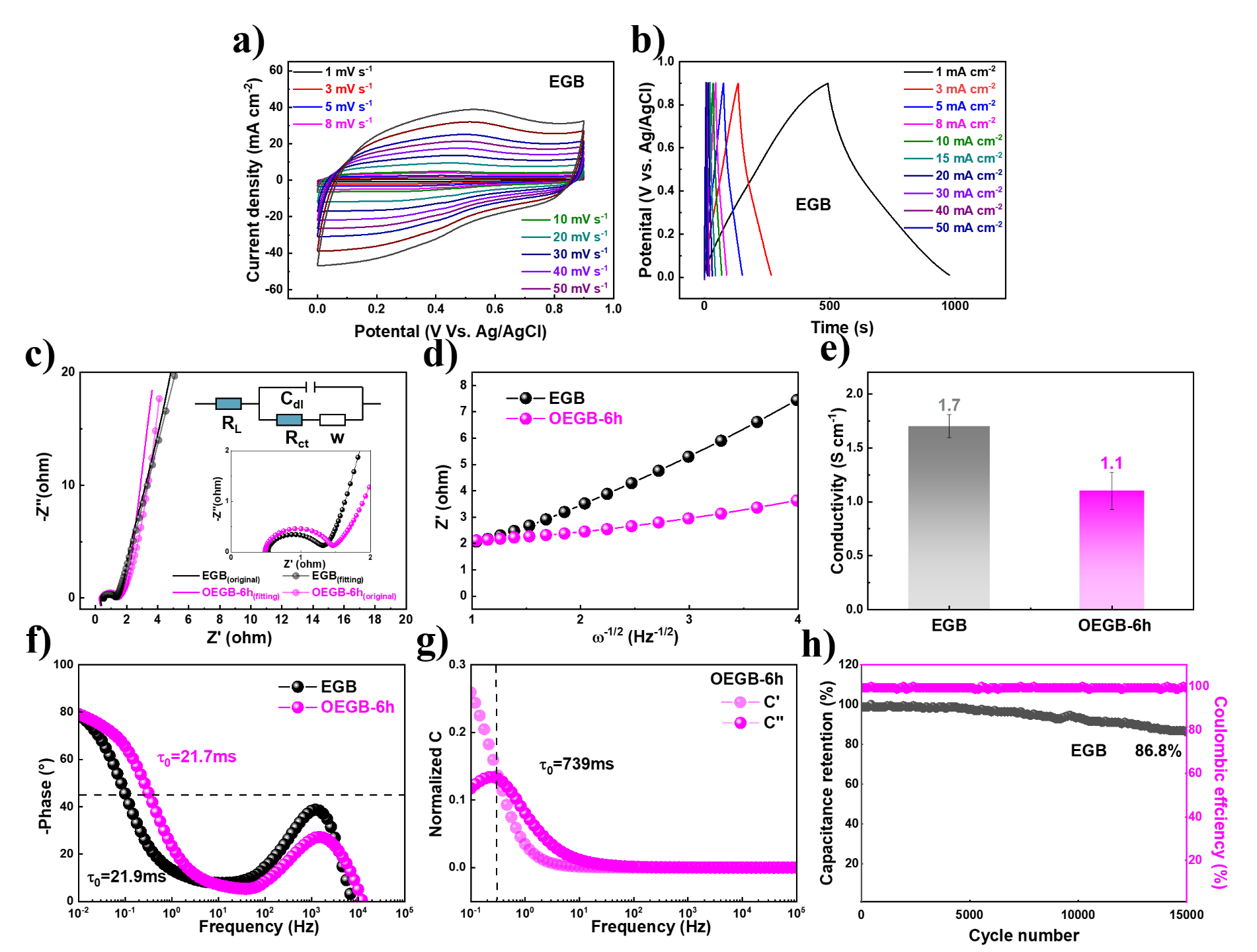


**Fig. S3** **a** CV curves of the EGB nanopaper at different scan rates; **b** GCD curves of the EGB nanopaper at various current densities; Theoretical analysis of the electrochemical behavior of the electrodes: **c** Nyquist impedance plots of the EGB and OEGB-6h nanopapers; **d** Linear fitting of Z′ versus ω^−1/2^, where a steeper slope indicates a lower ion diffusion coefficient; **e** electrical conductivity comparison of the samples EGB and OEGB; **f** Bode phase angle plots; **g** Frequency dependence of the imaginary capacitance; **h** Cycling stability and corresponding coulombic efficiency of the EGB nanopaper at 20 mA cm^–2^


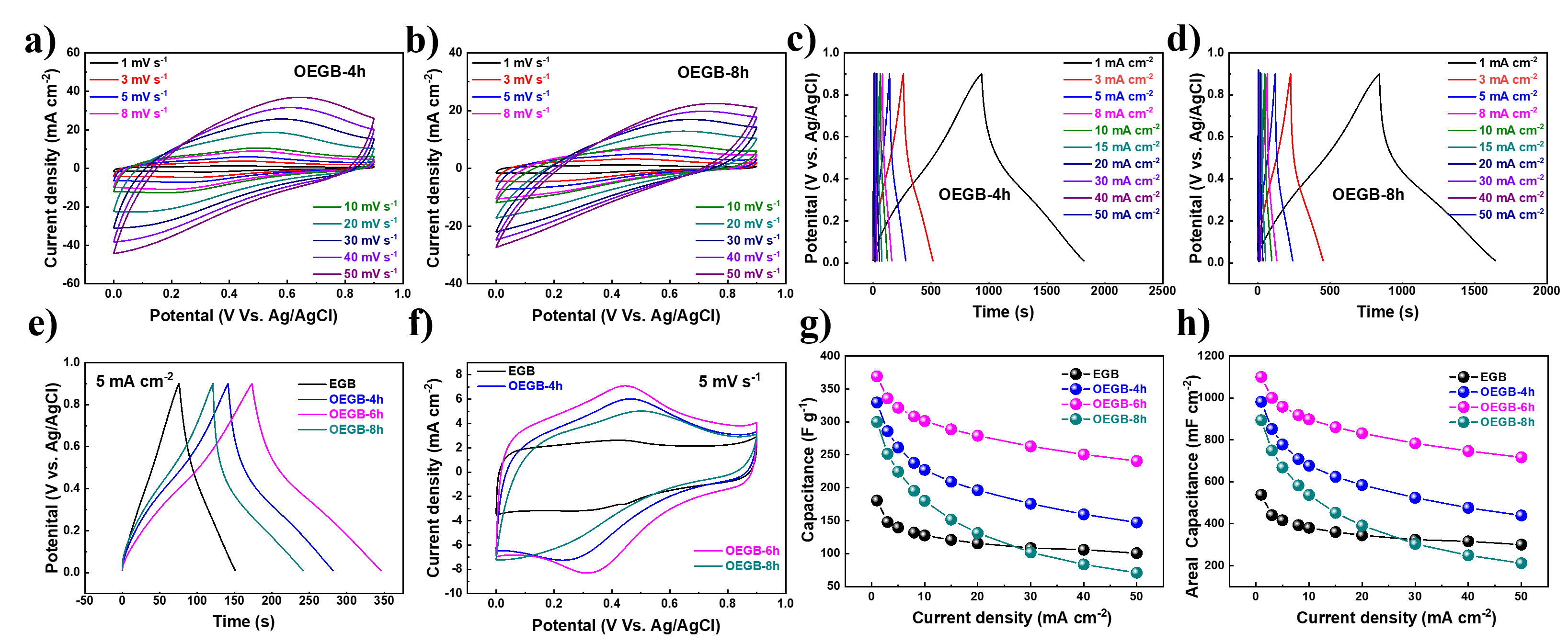


**Fig. S4** CV curves at different scan rates: **a** OEGB-4h nanopaper, **b** OEGB-8h nanopaper; GCD curves at various current densities: **c** OEGB-4h nanopaper, **d** OEGB-8h nanopaper; **e** GCD curves of EGB and OEGB nanopapers with different oxidation durations at a current density of 5 mA cm^-2^; **f** CV curves of OEGB nanopapers with different oxidation durations at a scan rate of 5 mV s^-1^; **g** Comparison of specific capacitance by mass for EGB and OEGB nanopapers; **h** Corresponding comparison of areal capacitance


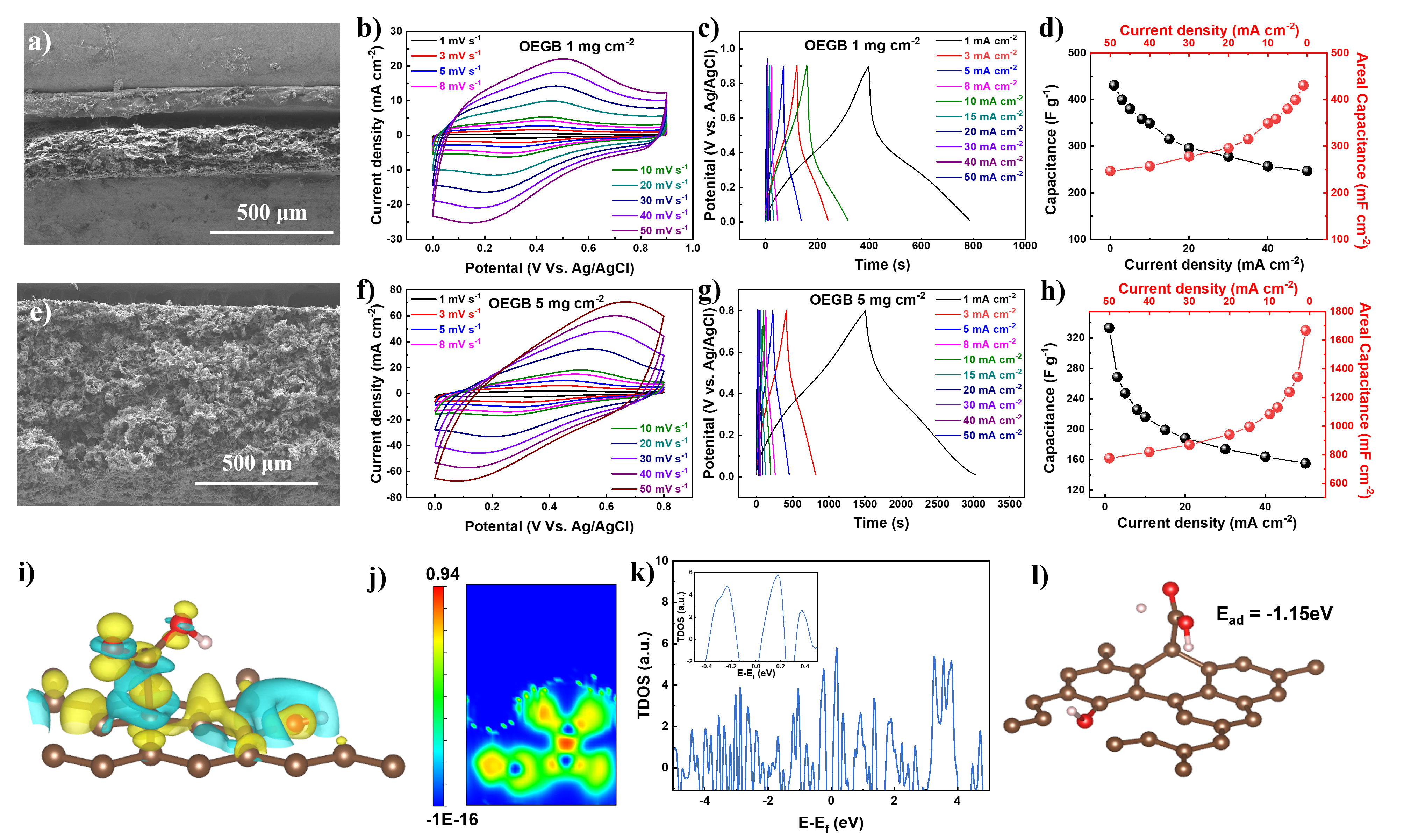


**Fig. S5** Cross-sectional SEM of OEGB under different mass loading: **a** 1 mg cm^-2^ and **e** 5 mg cm^-2^; Electrochemical performance of OEGB under a mass loading of 1 mg cm^-2^ and 5 mg cm^-2^: **b**, **f** CV curves at different scan rates; **c**, **g** GCD curves at various current densities; **d**, **h** Specific capacitance at current densities ranging from 1 to 50 mA cm^-2^; **i** Side views of differential charge density for -COOH:-OH=1:1; **j** Electron localization function (ELF) plots for -COOH:-OH=1:1; **k** Total density of states (DOS) at the Fermi level for -COOH:-OH=1:1; **l** Optimized structures of H+ adsorption on -COOH:-OH=1:1, with adsorption energies

**Table S2** Partial atomic charges in G-COOH calculated by Bader charge analysis (expressed in number of electrons; O (C-O) and O (C=O) represent the two oxygen atoms in the –COOH group)

|  | C | O (C-O) | O (C=O) | H |
| --- | --- | --- | --- | --- |
| G-COOH | -1.552 | 1.099 | 1.107 | -0.625 |


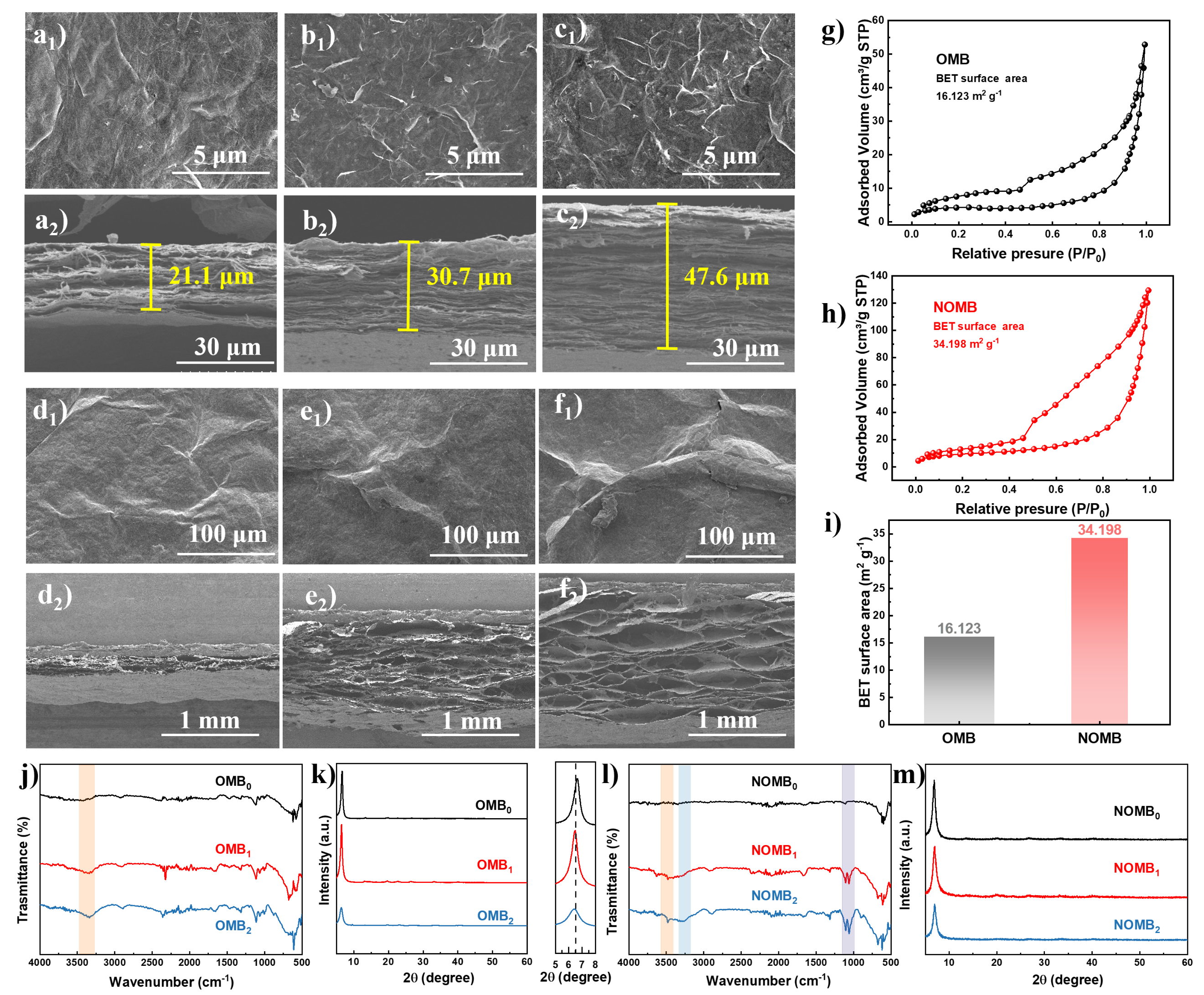


**Fig. S6** Morphology and chemical characterization of OMB and NOMB nanopapers. Surface SEM image of **a_1_** OMB_0_ nanopaper, **b_1_** OMB_1_ nanopaper, **c_1_** OMB_2_ nanopaper, **d_1_** NOMB_0_ nanopaper, **e_1_** NOMB_1_ nanopaper, **f_1_** NOMB_2_ nanopaper; Cross-sectional SEM image of **a_2_** OMB_0_ nanopaper, **b_2_** OMB_2_ nanopaper, **c_2_** OMB_2_ nanopaper, **d_2_** NOMB_0_ nanopaper, **e_2_** NOMB_1_ nanopaper, **f_2_** NOMB_2_ nanopaper; Nitrogen adsorption–desorption isotherms **g** OMB; **h** NOMB; **i** Comparison of specific surface areas of different samples; **j** OMB nanopaper of FTIR spectra; **k** OMB nanopaper of XRD spectra; **l** NOMB nanopaper of FTIR spectra, **m** NOMB nanopaper of XRD spectra


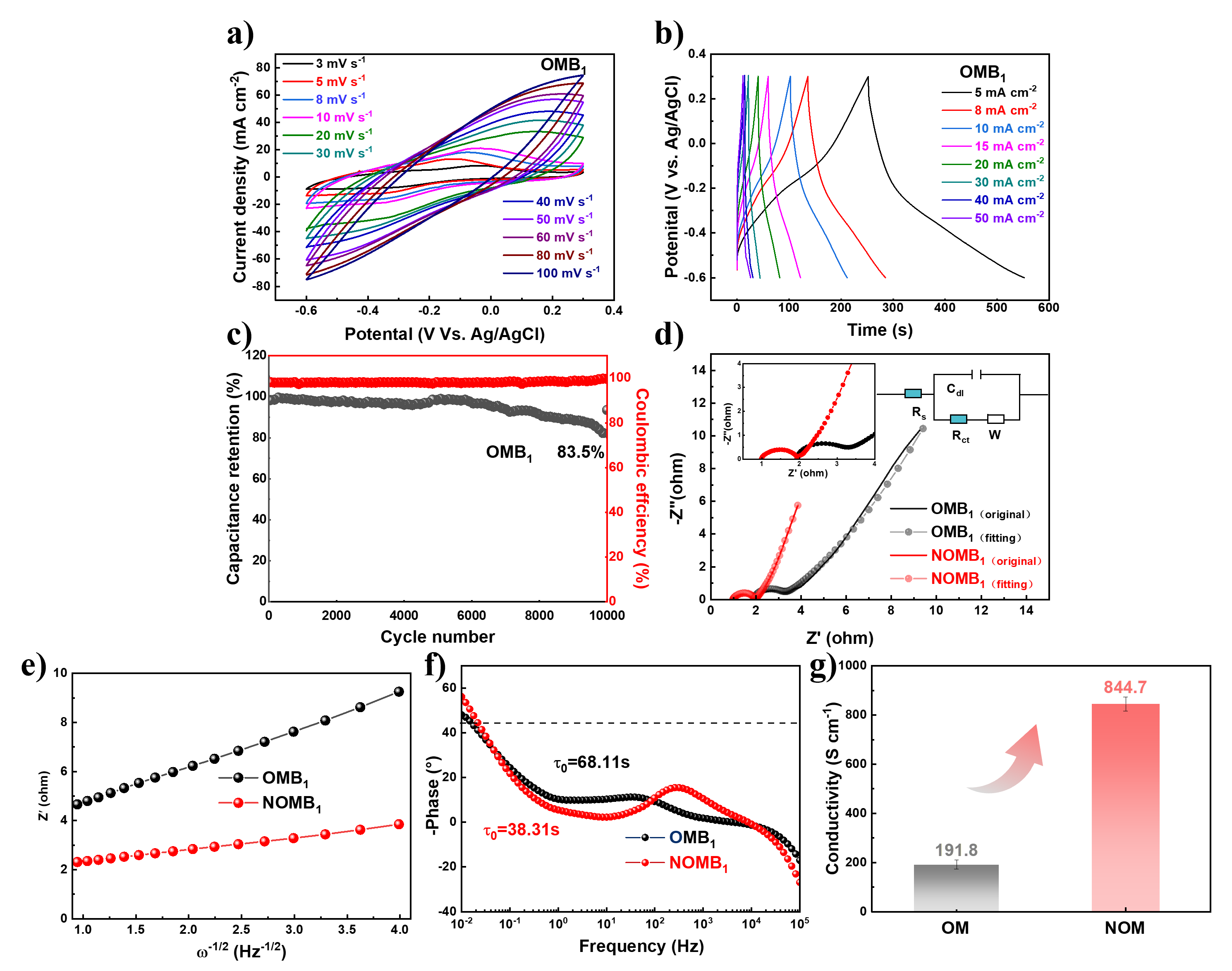


**Fig. S7** **a** CV curves of the OMB_1_ nanopaper at different scan rates; **b** GCD curves of the OMB_1_ nanopaper at various current densities; Theoretical analysis of the electrochemical behavior of the electrodes: **c** Cycling stability and corresponding coulombic efficiency of the OMB_1_ nanopaper at 20 mA cm^–2^**;** **d** Nyquist impedance plots of the OMB_1_ and NOMB_1_ nanopapers; **e** Linear fitting of Z′ versus ω^−1/2^, where a steeper slope indicates a lower ion diffusion coefficient; **f** Bode phase angle plots; **g** Electrical conductivity comparison of the samples before and after -NH_2_ functionalization


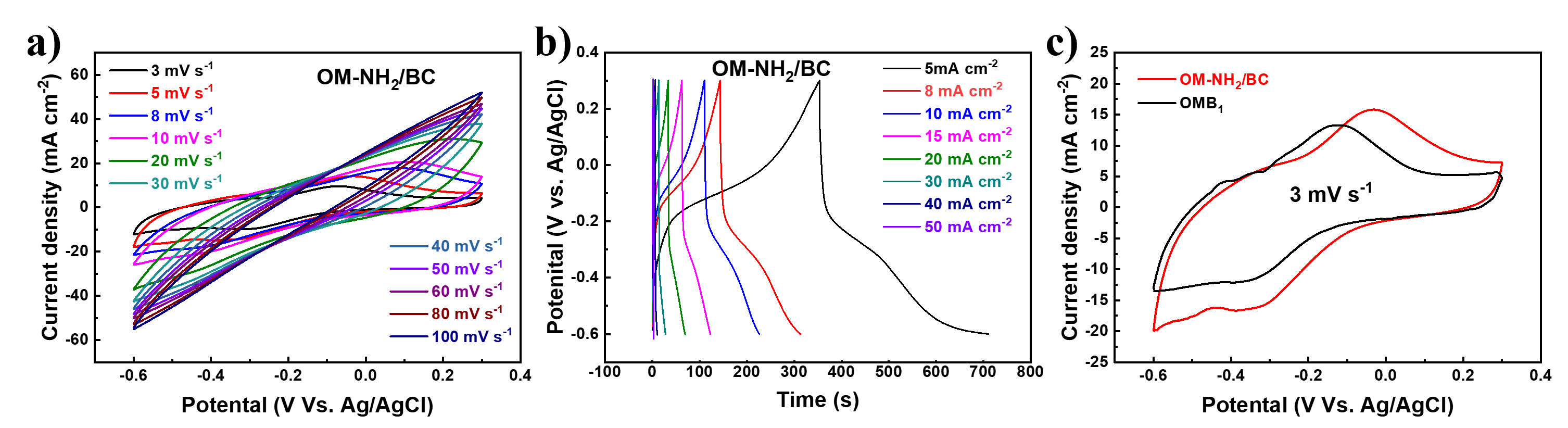


**Fig. S8** Electrochemical performance of OMB_1_ and OM-NH_2_/BC nanopaper. **a** CV curves of the OM-NH_2_/BC nanopaper at different scan rates; **b** GCD curves of the OM-NH_2_/BC nanopaper at various current densities; **c** CV curves of OM-NH_2_/BC nanopaper and OMB nanopaper at a current density of 5 mA cm^-2^


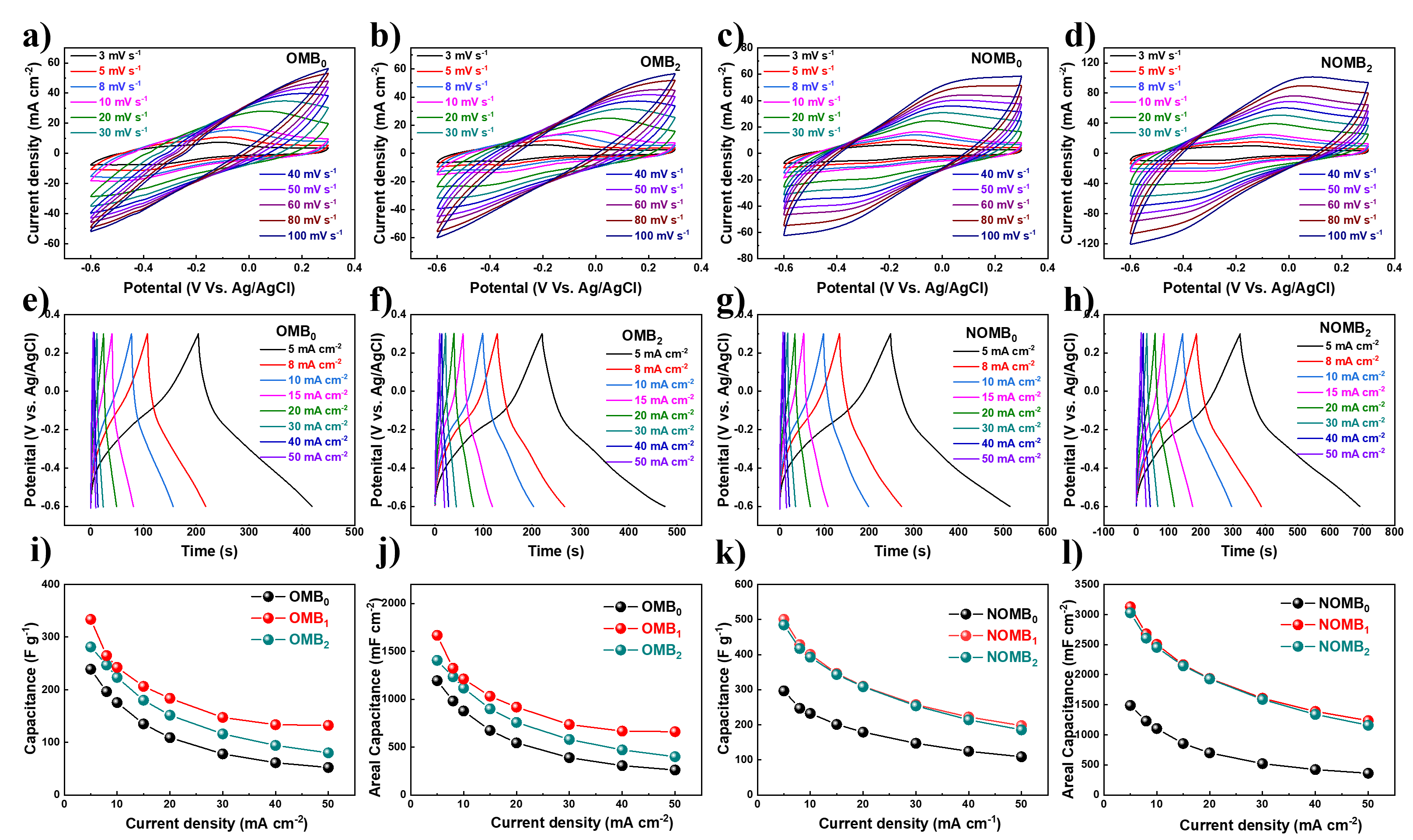


**Fig. S9** CV curves at different scan rates: **a** OMB_0_ nanopaper, **b** OMB_2_ nanopaper, **c** NOMB_0_ nanopaper, **d** NOMB_2_ nanopaper; GCD curves at a current density of 5 mA cm^-2^: **e** OMB_0_ nanopaper, **f** OMB_2_ nanopaper, **g** NOMB_0_ nanopaper, **h** NOMB_2_ nanopaper; **i** Comparison of specific capacitance by mass for OMB nanopapers; **j** Corresponding comparison of areal capacitance; **k** Comparison of specific capacitance by mass for NOMB nanopapers; **l** Corresponding comparison of areal capacitance

**
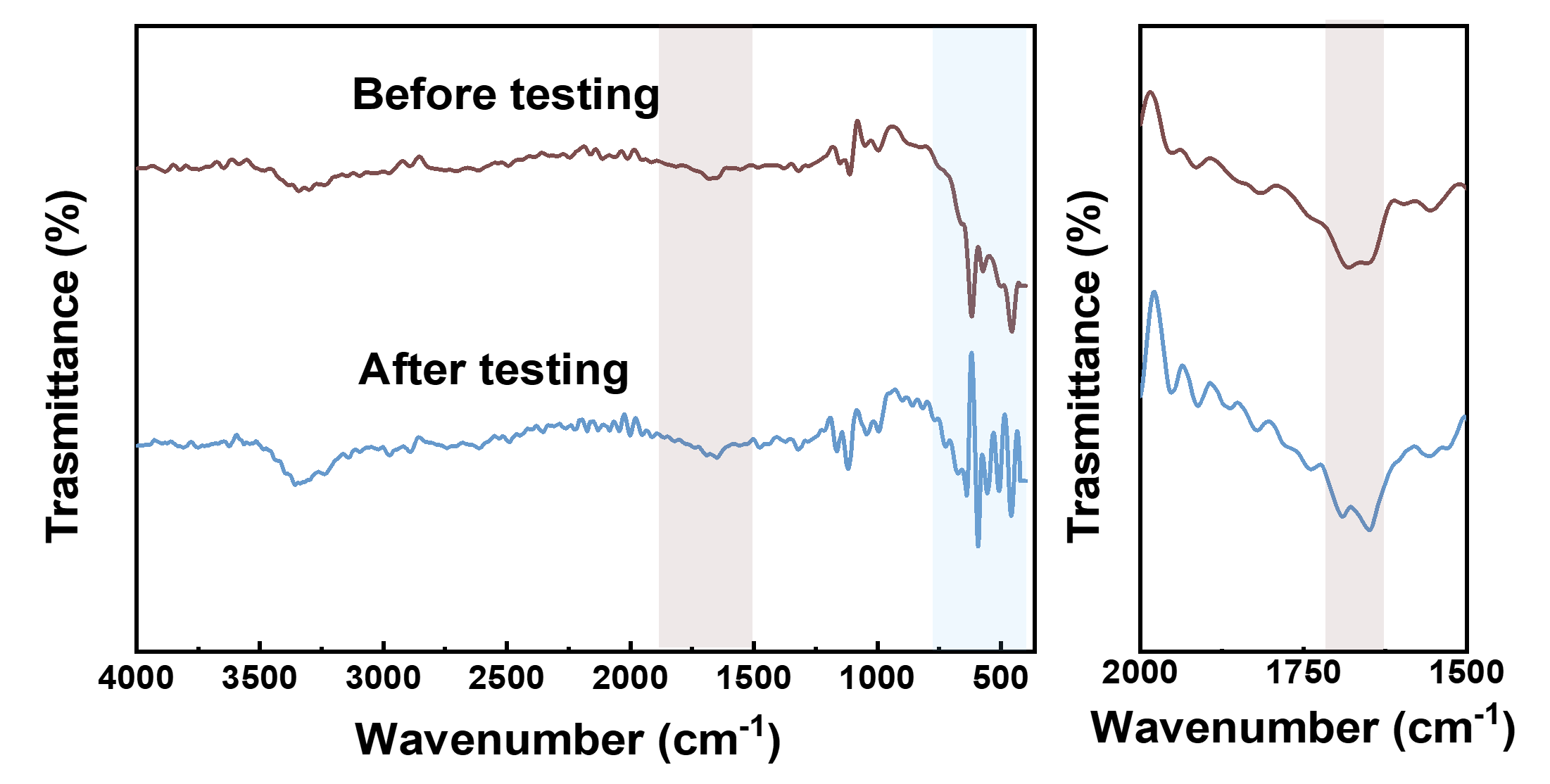
**

**Fig. S10** Comparison of the FTIR spectra of NOMB before and after electrochemical testing


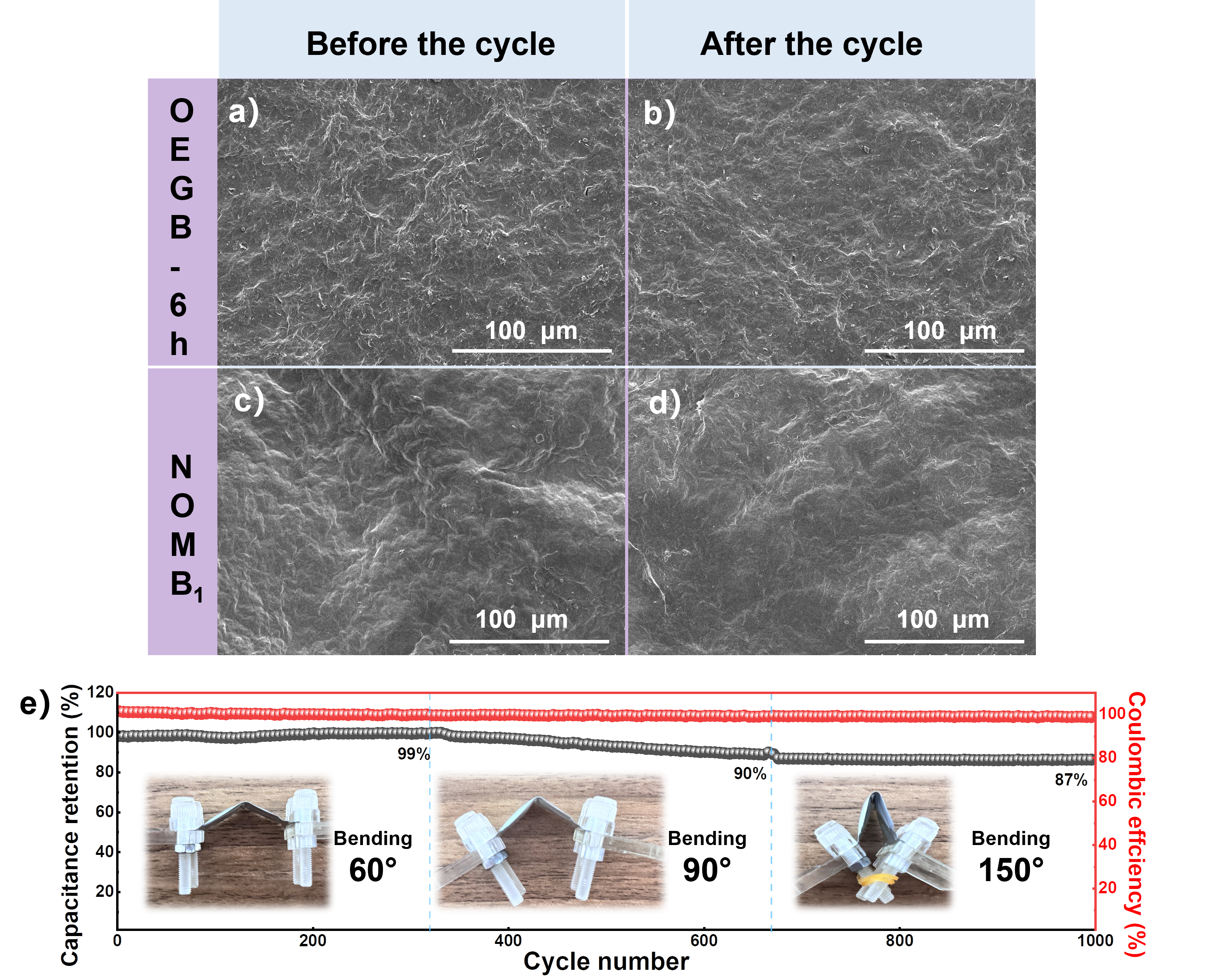


**Fig. S11** OEGB-6h before and after 30,000 charge-discharge cycles surface SEM images: **a** Before cycling; **b** After cycling; NOMB_1_ before and after 10,000 charge-discharge cycles surface SEM images: **c** Before cycling; **d** After cycling; **e** After undergoing bending at different angles and 1000 charge-discharge cycles, the ACS device still maintains 87% of its capacity

| Graphene -based  electrode | Electrolyte | Mass loading | Test condition | Cg  (F g^-1^) | Ca  (mF cm^-2^) | Refs. |
| --- | --- | --- | --- | --- | --- | --- |
| MXene derived TiO_2_@rGO | 1M H_2_SO_4_ |  | 5 mA cm^-2^ |  | 662.9 | [S1] |
| RGO | 1M H_2_SO_4_ | 2.2 mg cm^-2^ | 0.1 A g^-1^ | 314 | 609 | [S2] |
| MXene/GO | 1M H_2_SO_4_ | 1.15 mg cm^-2^ | 1 mV s^-1^ | 228.4 | 198.8 | [S3] |
| rGO/WO_3_ | 1M H_2_SO_4_ |  | 0.3 A g^-1^ | 287 |  | [S4] |
| FL-MoS_2_@rGO-2 | 1M H_2_SO_4_ | 1.5 mg cm^-2^ | 1 A g^-1^ | 346.1 | 519.6 | [S5] |
| DNQ/rGO/PVA-2 | PVA/H_2_SO_4_ |  | 1 mA cm^-2^ |  | 618.2 | [S6] |
| VC/GO | 1M H_2_SO_4_ |  | 1 A g^-1^ | 174 |  | [S7] |
| GO/EG | EMIMBF4 | 1 mg cm^-2^ | 1 A g^-1^ | 236 | 215 | [S8] |
| MnO-Mn_3_O_4_/MnS/LIG | 1M LiCl |  | 1 mA cm^-2^ |  | 741.6 | [S9] |
| Mo_3_C_2_/LIG | 1M H_2_SO_4_ | 5 mg cm^-2^ | 0.5 A g^-1^ | 62 | 11.9 | [S10] |
| OEG-6h | 1M H_2_SO_4_ | 1 mg cm^-2^ | 1 mA cm^-2^ | 430.5 | 430 | This work |
| OEG-6h | 1M H_2_SO_4_ | 3 mg cm^-2^ | 1 mA cm^-2^ | 369.1 | 1107.3 | This work |
| OEG-6h | 1M H_2_SO_4_ | 5 mg cm^-2^ | 1 mA cm^-2^ | 333.6 | 1668 | This work |

**Table S3** Comparison of electrochemical performance of OEGB-6h electrode with other advanced graphene-based electrodes

**Table S4** Comparison of electrochemical performance of NOMB_1_ electrode with other advanced MXene-based electrodes

| MXene-based  electrode | Electrolyte | Mass loading | Test condition | Cg  (F g^-1^) | Ca  (mF cm^-2^) | Refs. |
| --- | --- | --- | --- | --- | --- | --- |
| MXene/AC_3_ | PVA/H_2_  SO_4_ |  | 5 mV s^-1^ | 407 |  | [S11] |
| MXene/BC | 3M H_2_SO_4_ | 8 mg cm^-2^ | 3 mV s^-1^ | 416 | 2084 | [S12] |
| MXene/HLNPs | 1M H_2_SO_4_ | 5 mg cm^-2^ | 1 A g^-1^ | 241 | 1273 | [S13] |
| Ti_3_C_2_Tx@MPS | 3M H_2_SO_4_ | 4 mg cm^-2^ | 5 mV s^-1^ | 469 | 2620 | [S14] |
| PLA/PANI/MXene | 1M H_2_SO_4_ | 3 mg cm^-2^ | 1 A g^-1^ | 290.8 | 872.4 | [S15] |
| MXene derived TiO_2_@rGO | 1M H_2_SO_4_ |  | 5 mA cm^-2^ |  | 662.9 | [S1] |
| DAAQ-COFs/ Ti_3_C_2_T_x_ | 1M H_2_SO_4_ |  | 1 A g^-1^ | 361 |  | [S16] |
| MXene/rGO/CNT | PVA/H_2_SO_4_ | 5 mg cm^-2^ | 1 A g^-1^ | 463.5 | 2317.5 | [S17] |
| CMC/Ti_3_C_2_T_x_/ZIF-67-2:3 | 3M KOH |  | 1 A g^-1^ | 481.7 |  | [S18] |
| Ti_3_C_2_T_x_/GDY-NTs | 1M H_2_SO_4_ |  | 2 A g^-1^ | 337.4 |  | [S19] |
| NOMB_1_ | 1M H_2_SO_4_ | 5 mg cm^-2^ | 5 mA cm^-2^ | 500.5 | 2502.5 | This work |

**Table S5** Comparison of the electrochemical performance with other graphene or MXene-based assembled supercapacitors

|  | Electrolyte | Cg  (F g^-1^) | Ca  (mF cm^-2^) | Energy density (Wh kg ^-1^) | Power density (W kg^-1^) | Cycle stability | Refs. |
| --- | --- | --- | --- | --- | --- | --- | --- |
| rGO//Ti_3_C_2_Tx | 1M  H_2_SO_4_ | 48 |  | 8 | 50 | For 1000 cycles, 76% | [S20] |
| MCN/CESe5:1//Ti_3_C_2_T_x_ | PVA/KOH | 60 |  | 10.1 | 2203.6 | For 10000 cycles, 100% | [S21] |
| MXene/rGO//MXene/rGO | PVA/H_2_SO_4_ | 6 |  | 3.81 | 163 | For 10000 cycles, 94.5% | [S22] |
| MXene/BC//MXene/BC | 3M  H2SO4 |  |  | 9.63 | 250 | - | [S23] |
| MXene-MnO_2_//MnO_2_/CF | PVA/H_2_SO_4_ | 20.5 | 81.0 | 6.4 | 2300 | For 3000 cycles, 84% | [S24] |
| Ti_3_C_2_T_x_// CFP | 1M KOH | 20.1 |  | 4.5 | 1300 | For 15000 cycles, 94.4% | [S25] |
| MXene/HLNPs//MXene/HLNPs | PVA/H_2_SO_4_ | 95 | 514 |  |  | For 5000 cycles, 82.0% | [S13] |
| PLA/PANI/MXene//PLA/PANI/MXene | PVA/H_2_SO_4_ |  |  | 9.3 | 291.3 | For 5000 cycles, 80.3% | [S15] |
| WO_3_/rGO //activated carbon | PVA/H_2_SO_4_ | 139 |  | 19.1 | 432 | For 5000 cycles, 81.3% | [S26] |
| MnO-Mn_3_O_4_/MnS/LIG//MnO-Mn_3_O_4_/MnS/LIG | LiCl |  | 170.4 |  |  | For 6000 cycles, 87.6% | [S9] |
| MXene/rGO | H_2_SO_4_ | 335.3 | 1039.7 | 10.5 | 80.3 | For 20000 cycles, 60.9% | [S27] |
| NOMB//OEGB | H_2_SO_4_/AM | 188.4 | 1570 | 58.9 | 3802 | For 30000 cycles, 89.7% | This work |

**Table S6** Comparison of the electrochemical performance, especially the volumetric electrochemical performance, with other graphene or MXene-based assembled supercapacitors

|  | Electrolyte | Ca  (F cm^-3^) | Energy density (mWh cm ^-3^) | Power density (mW cm^-3^) | Method | Refs.  (DOI) |
| --- | --- | --- | --- | --- | --- | --- |
| HNHG–PANI// HNHG–PANI | H_2_SO_4_ | 1058 |  |  | Hydrothermal followed by polymerization | [S28] |
| NS-GF// NS-GF | PVA/H_2_SO_4_ | 59.9 | 8.3 | 1048.4 | Template | [S29] |
| RGO/CNT@CMC//RGO/CNT@CMC | PVA/H_3_PO_4_ | 39.5 | 3.5 | 18 | Coaxial spinning | [S30] |
| RGO/CNT//RGO/CNT | PVA/H_3_PO_4_ | 38.8 | 3.4 | 700 | Mix | [S31] |
| Ti_3_C_2_T_x_  Mn//Ti_3_C_2_  T_x_-Mn | PVA/H_2_SO_4_ |  | 52.4 | 1300 | Metal ion-induced | [S32] |
| Ti_3_C_2_T_x_//T i_3_C_2_T_x_ | PVA/H_2_SO_4_ | 439 | 23.3 | 300 | In-situ intercalation | [S33] |
| NiCo_2_S_4_-13@W-MX/CF-4//NiCo_2_S_4_-13@W-MX/CF-4 | KOH | 275.9 | 40 | 301.5 | electro-deposition | [S34] |
| MXene/rGO//MXene/rGO | H_2_SO_4_ | 341 | 5.1 | 1700 | Mix | [S35] |
| MXene/rGO//MXene/rGO | Na_2_SO_4_ |  | 135 | 400 | laser-induce | [S36] |
| NOMB//OEGB | H_2_SO_4_/AM | 1207.7 | 452.8 | 29252.4 | Gas expansion | This work |

**Supplementary References**

1. S. Park, S.H. Choi, J.M. Kim, S. Ji, S. Kang et al., Nanoarchitectonics of MXene derived TiO_2_/graphene with vertical alignment for achieving the enhanced supercapacitor performance. Small **20**(6), e2305311 (2024). <https://doi.org/10.1002/smll.202305311>
2. J. Tan, Z. Liu, W. Wu, G. Li, W. Guo, Reduced graphene oxides prepared *via* mass loading-controlled non-explosive thermal reduction for high volumetric capacitance supercapacitors. J. Mater. Chem. A **13**(16), 11330–11343 (2025). <https://doi.org/10.1039/d5ta01172h>
3. X. Wei, J. Wang, R. Chen, Z. Zhang, Q. Zhang, Large capacitance enhancement achieved *via* tuning electrode/electrolyte thickness of graphene oxide-based all-solid-state supercapacitors. Small **21**(22), e2501802 (2025). <https://doi.org/10.1002/smll.202501802>
4. J.C. Pieretti, T.B. Trevisan, M.M.M. de Moraes, E.A. de Souza, S.H. Domingues, High capacitive rGO/WO_3_ nanocomposite: the simplest and fastest route of preparing it. Appl. Nanosci. **10**(1), 165–175 (2020). <https://doi.org/10.1007/s13204-019-01089-z>
5. Y. Zhang, J. Xu, S. Lu, H. Li, T. Yonar et al., Engineering few-layer MoS_2_ and rGO heterostructure composites for high-performance supercapacitors. Adv. Compos. Hybrid Mater. **8**, 108 (2025). <https://doi.org/10.1007/s42114-024-01159-z>
6. Z. Li, G. Chen, H. Yang, C. Meng, Z. Zhang et al., Development of a 2, 3-dichloro-1, 4-naphthoquinone/polyvinyl alcohol composite material and its application in all-solid-state flexible supercapacitors. Chem. Eng. J. **507**, 160803 (2025). <https://doi.org/10.1016/j.cej.2025.160803>
7. T. Lin, X. Ren, X. Wen, A. Karton, V. Quintano et al., Membrane based *In-situ* reduction of graphene oxide for electrochemical supercapacitor application. Carbon **224**, 119053 (2024). <https://doi.org/10.1016/j.carbon.2024.119053>
8. C. Li, X. Li, Q. Yang, L. Wu, C. Wang et al., Vascular system inspired 3D electrolyte network for high rate and high mass loading graphene supercapacitor. Adv. Funct. Mater. **34**(26), 2315137 (2024). <https://doi.org/10.1002/adfm.202315137>
9. Y. Song, N. Li, J. Kang, Z. Li, N. Hong et al., Heterostructure-anchored 3D CNT-bridged graphene architecture *via* layer-by-layer structural engineering for thick electrodes of supercapacitors. Chem. Eng. J. **497**, 154557 (2024). <https://doi.org/10.1016/j.cej.2024.154557>
10. C. Yang, X. Zhou, Y. Tian, J. Zhu, M. Xiao et al., One-Step laser induced molybdenum carbide/graphene hybrid electrode for supercapacitor. Chem. Eng. J. **499**, 156519 (2024). <https://doi.org/10.1016/j.cej.2024.156519>
11. R. Sinha, P.S. Kiran, K.V. Kumar, N. Pandit, C. Satish et al., MXene/Biomass-derived activated carbon composite for supercapacitor applications. Carbon **236**, 120101 (2025). <https://doi.org/10.1016/j.carbon.2025.120101>
12. Y. Wang, X. Wang, X. Li, Y. Bai, H. Xiao et al., Engineering 3D ion transport channels for flexible MXene films with superior capacitive performance. Adv. Funct. Mater. **29**(14), 1900326 (2019). <https://doi.org/10.1002/adfm.201900326>
13. H. Zhang, C. Hao, T. Fu, D. Yu, J. Howe et al., Gradient-layered MXene/hollow lignin nanospheres architecture design for flexible and stretchable supercapacitors. Nano-Micro Lett. **17**(1), 43 (2024). <https://doi.org/10.1007/s40820-024-01512-3>
14. Y. Song, Y. Liu, Q. Ao, L. Jiang, X. Lv et al., Efficient utilization of the active sites of MXene: MXene/PSSNa films with a 3D-stabilized porous structure as high-capacitance and high-rate electrodes for flexible supercapacitors. J. Mater. Chem. A **12**(42), 29230–29240 (2024). <https://doi.org/10.1039/d4ta05925e>
15. Z. Li, J. Li, B. Wu, H. Wei, H. Guo et al., Interfacial-engineered robust and high performance flexible polylactic acid/polyaniline/MXene electrodes for high-perfarmance supercapacitors. J. Mater. Sci. Technol. **203**, 201–210 (2024). <https://doi.org/10.1016/j.jmst.2024.02.084>
16. N. An, Z. Guo, C. Guo, M. Wei, D. Sun et al., A novel COF/MXene film electrode with fast redox kinetics for high-performance flexible supercapacitor. Chem. Eng. J. **458**, 141434 (2023). <https://doi.org/10.1016/j.cej.2023.141434>
17. W. Luo, Q. Liu, B. Zhang, J. Li, R. Li et al., Binder-free flexible Ti_3_C_2_T*_x_* MXene/reduced graphene oxide/carbon nanotubes film as electrode for asymmetric supercapacitor. Chem. Eng. J. **474**, 145553 (2023). <https://doi.org/10.1016/j.cej.2023.145553>
18. H. Xu, Q. Hu, T. Zhao, J. Zhu, Z. Lian et al., Sodium carboxymethylcellulose/MXene/zeolite imidazolium framework-67-derived 3D porous carbon aerogel for high-performance asymmetric supercapacitors. Carbohydr. Polym. **326**, 121641 (2024). <https://doi.org/10.1016/j.carbpol.2023.121641>
19. Y. Wang, N. Chen, Y. Liu, X. Zhou, B. Pu et al., MXene/Graphdiyne nanotube composite films for Free-Standing and flexible Solid-State supercapacitor. Chem. Eng. J. **450**, 138398 (2022). <https://doi.org/10.1016/j.cej.2022.138398>
20. A.M. Navarro-Suárez, K.L. Van Aken, T. Mathis, T. Makaryan, J. Yan et al., Development of asymmetric supercapacitors with titanium carbide-reduced graphene oxide couples as electrodes. Electrochim. Acta **259**, 752–761 (2018). <https://doi.org/10.1016/j.electacta.2017.10.125>
21. S. De, J. Florentino, G. Pathiraja, B.R. Gautam, B.P. Bastakoti, MXene/graphitic carbon nitride-supported metal selenide for all-solid-state flexible supercapacitor and oxygen evolution reaction. J. Mater. Chem. A **13**(16), 11300–11313 (2025). <https://doi.org/10.1039/d4ta08907c>
22. D. Jiang, J. Zhang, S. Qin, Z. Wang, K.A.S. Usman et al., Superelastic Ti_3_C_2_T*_x_* MXene-based hybrid aerogels for compression-resilient devices. ACS Nano **15**(3), 5000–5010 (2021). <https://doi.org/10.1021/acsnano.0c09959>
23. Y. Luo, W. Que, Y. Tang, Y. Kang, X. Bin et al., Regulating functional groups enhances the performance of flexible microporous MXene/bacterial cellulose electrodes in supercapacitors. ACS Nano **18**(18), 11675–11687 (2024). <https://doi.org/10.1021/acsnano.3c11547>
24. Y. Wei, M. Zheng, W. Luo, B. Dai, J. Ren et al., All pseudocapacitive MXene-MnO_2_ flexible asymmetric supercapacitor. J. Energy Storage **45**, 103715 (2022). <https://doi.org/10.1016/j.est.2021.103715>
25. W. Wu, S. Lin, T. Chen, L. Li, Y. Pan et al., Performance evaluation of asymmetric supercapacitor based on Ti_3_C_2_T*_x_*-paper. J. Alloys Compd. **729**, 1165–1171 (2017). <https://doi.org/10.1016/j.jallcom.2017.09.256>
26. S.B. Patil, R.P. Nikam, V.C. Lokhande, C.D. Lokhande, R.S. Patil, Tungsten oxide/reduced graphene oxide composite electrodes for solid-state asymmetric supercapacitor application. Adv. Compos. Hybrid Mater. **8**(2), 175 (2025). <https://doi.org/10.1007/s42114-025-01268-3>
27. J. Yan, C.E. Ren, K. Maleski, C.B. Hatter, B. Anasori et al., Flexible MXene/graphene films for ultrafast supercapacitors with outstanding volumetric capacitance. Adv. Funct. Mater. **27**(30), 1701264 (2017). <https://doi.org/10.1002/adfm.201701264>
28. Z. Fan, Z. Cheng, J. Feng, Z. Xie, Y. Liu et al., Ultrahigh volumetric performance of a free-standing compact N-doped holey graphene/PANI slice for supercapacitors. J. Mater. Chem. A **5**(32), 16689–16701 (2017). <https://doi.org/10.1039/c7ta04384h>
29. W. Ma, W. Li, M. Li, Q. Mao, Z. Pan et al., Scalable microgel spinning of a three-dimensional porous graphene fiber for high-performance flexible supercapacitors. J. Mater. Chem. A **8**(47), 25355–25362 (2020). <https://doi.org/10.1039/d0ta08937k>
30. L. Kou, T. Huang, B. Zheng, Y. Han, X. Zhao et al., Coaxial wet-spun yarn supercapacitors for high-energy density and safe wearable electronics. Nat. Commun. **5**, 3754 (2014). <https://doi.org/10.1038/ncomms4754>
31. Y. Ma, P. Li, I. Sedloff, X. Zhang, H. Zhang et al., Conductive graphene fibers for wire-shaped supercapacitors strengthened by unfunctionalized few-walled carbon nanotubes. ACS Nano **9**(2), 1352–1359 (2015). <https://doi.org/10.1021/nn505412v>
32. H. Xu, J. Fan, H. Su, C. Liu, G. Chen et al., Metal ion-induced porous MXene for all-solid-state flexible supercapacitors. Nano Lett. **23**(1), 283–290 (2023). <https://doi.org/10.1021/acs.nanolett.2c04320>
33. S. Yang, P. Zhang, F. Wang, A.G. Ricciardulli, M.R. Lohe et al., Fluoride-free synthesis of two-dimensional titanium carbide (MXene) using a binary aqueous system. Angew. Chem. Int. Ed. **57**(47), 15491–15495 (2018). <https://doi.org/10.1002/anie.201809662>
34. D.D. Khumujam, T. Kshetri, T.I. Singh, N.H. Kim, J.H. Lee, Fibrous asymmetric supercapacitor based on wet spun MXene/PAN Fiber-derived multichannel porous MXene/CF negatrode and NiCo2S4 electrodeposited MXene/CF positrode. Chem. Eng. J. **449**, 137732 (2022). <https://doi.org/10.1016/j.cej.2022.137732>
35. S. Seyedin, E.R.S. Yanza, J.M. Razal, Knittable energy storing fiber with high volumetric performance made from predominantly MXene nanosheets. J. Mater. Chem. A **5**(46), 24076–24082 (2017). <https://doi.org/10.1039/c7ta08355f>
36. S. Gupta, M. Narajczyk, M. Sawczak, J.B. Jasinski, R. Bogdanowicz et al., Flexible MXene/laser-induced porous graphene asymmetric supercapacitors: enhanced energy density of lateral and sandwich architectures under different electrolytes. Small **21**(20), 2502297 (2025). <https://doi.org/10.1002/smll.202502297>
